# The BCL9-2 proto-oncogene governs estrogen receptor alpha expression in breast tumorigenesis

**DOI:** 10.18632/oncotarget.2252

**Published:** 2014-07-25

**Authors:** Nathalie Zatula, Maria Wiese, Jens Bunzendahl, Walter Birchmeier, Christina Perske, Annalen Bleckmann, Felix H. Brembeck

**Affiliations:** ^1^ Tumor Biology and Signal Transduction, Georg-August-University Göttingen, Germany; ^2^ Max-Delbrueck-Center for Molecular Medicine, Berlin, Germany; ^3^ Dept. of Pathology, Georg-August-University Göttingen, Germany; ^4^ Dept. of Hematology and Medical Oncology, Georg-August-University Göttingen, Germany

**Keywords:** mouse model, primary cell culture, human breast cancer, canonical Wnt signaling, estrogen receptor pathway, Pygo2, Sp1

## Abstract

The majority of human breast cancers express estrogen receptor alpha (ER), which is important for therapy with anti-estrogens. Here we describe the role of BCL9-2, a proto-oncogene previously characterized as co-activator of Wnt/ß-catenin signaling, for mammary tumorigenesis in mice and human. ER positive human breast cancers showed overexpression of BCL9-2 and tamoxifen treated patients with high BCL9-2 demonstrated a better survival. BCL9-2 was upregulated during puberty and pregnancy in normal mammary epithelia, but downregulated in the involuted gland. BCL9-2 overexpression *in vivo* delayed the mammary involution and induced alveolar hyperplasia. Moreover, aged BCL9-2 transgenic mice developed ductal-like mammary tumors with high nuclear ER expression. We found, that primary cell cultures of BCL9-2 breast tumors responded to tamoxifen treatment. Moreover, BCL9-2 regulated the expression of ER and the proliferation of human breast cancer cells independently of ß-catenin. Finally, we describe a novel mechanism, how BCL9-2 regulates ER transcription by interaction with Sp1 through the proximal ESR1 gene promoter. In summary, BCL9-2 induces ER positive breast cancers *in vivo*, regulates ER expression by a novel ß-catenin independent mechanism in breast cancer cells, and might predict the therapy response to tamoxifen treatment.

## INTRODUCTION

Expression of nuclear estrogen receptor alpha (ER) predicts the prognosis and the therapy response to anti-estrogens of human breast cancer [1]. Upon binding of the estrogen ligand, the receptor is translocated to the nucleus and binds to the DNA of target genes that promote tumorigenesis but also regulate transcription of the receptor itself [1]. In fact, regulation of ER expression is modulated on the transcriptional level, by protein modification or degradation [2; 3]. However, the molecular mechanisms that maintain ER expression in breast cancer are not well understood [1]. Amplifications of the ESR1 gene (which encodes ER) are only found in up to 20% of breast cancers [2]. In contrast, transcriptional regulation of the ESR1 gene promoter was described in a number of studies to be an important mechanism for overexpression and maintenance of ER expression in breast tumors [1]. One of the most important regulators of ER transcription is Sp1 (specificity protein 1) that is overexpressed in human ER positive (ER+) breast cancers [4]. Sp1 binds to several G/C-rich elements located within the proximal promoter and initiates the transcription of ER in breast cancer cells together with several co-factors of the basal transcription machinery [5-7].

So far, only few genetic mouse models have been established that recapitulate human ER+ breast cancers [8]. Most animal models, including mutants for components of canonical Wnt-signaling, develop ER negative breast cancers [8]. The Wnt/ß-catenin pathway controls stem cell maintenance and differentiation in different organs, and aberrant activation of canonical Wnt-signaling is also implicated in breast cancer [9; 10]. The signaling activity of the Wnt/ß-catenin pathway is modulated by the members of the Legless/B-Cell Lymphoma 9 (*Lgs/BCL9*) and *Pygopus* gene families [11-14]. The BCL9 and Pygopus co-factors establish a nuclear complex with ß-catenin/Lef/Tcf's and regulate the transcriptional output of the pathway. In contrast to the essential function of the Drosophila *Lgs/BCL9* and *Pygopus* genes for Wnt signaling during embryonic development, the mammalian orthologs act as specific co-factors to enhance Wnt activity beyond a certain threshold during embryogenesis and tumorigenesis [15-17]. We have previously characterized the proto-oncogene BCL9-2 (*BCL9L*, B-Cell Lymphoma 9–like) which augments Wnt/ß-catenin signaling and promotes intestinal tumor progression [11; 15; 18]. Moreover, recent data indicated that the function of BCL9 and Pygopus proteins is apparently not limited to the canonical Wnt pathway in mice and human [14; 15; 17].

So far, the role of de-regulated Wnt-signaling in breast tumorigenesis is controversial, since activating mutations of ß-catenin or loss of the Adenomatous Polyposis Coli (APC) gene product are uncommon in human breast cancer [19]. In general, hyperactivated Wnt/ß-catenin signaling in human and mice is linked to basal-like breast cancers that are hormone receptor negative [19]. For instance, genetic mouse mutants with truncated APC, stabilized ß-catenin or overexpression of Wnt10b develop basal-like, triple-negative breast cancers and show in part squamous metaplasia, which is unusual for human breast cancer [20-24]. In contrast, activation of the Wnt1 proto-oncogene results in the formation of ER+ ductal-like breast cancers, presumably by Wnt/ß-catenin independent mechanisms [25-27].

Here we describe a novel ß-catenin independent role of the nuclear co-factor BCL9-2 for the development of ER+ breast cancers. We report, that BCL9-2 overexpression *in vivo* leads to premalignant alterations in the breast and induces mammary tumors in aged mice with high nuclear ER expression that resemble ductal-like human breast cancers. Mechanistically, we provide evidence that BCL9-2 transcriptionally regulates ER expression in breast cancer cells independently of ß-catenin. We identify a novel interaction with Sp1, which controls ER expression though transcriptional regulation of the proximal ESR1 gene promoter. Our results are of clinical significance, since we show that BCL9-2 is also highly expressed in ER+ human breast cancers and might predict the therapy response of tamoxifen treated breast cancer patients.

## RESULTS

### BCL9-2 is highly upregulated in human ER+ breast cancers and might be predictive for the response to tamoxifen therapy

**Figure 1:**
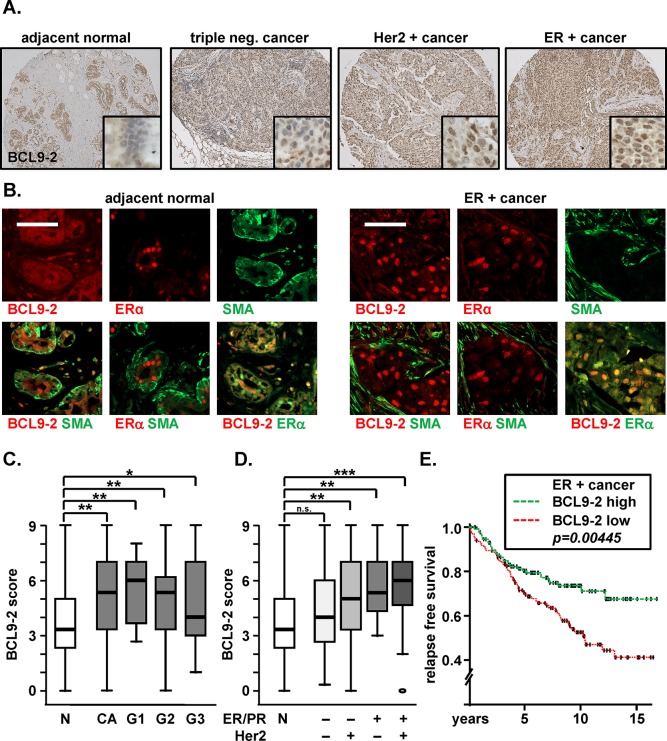
BCL9-2 is highly expressed in human ER+ breast cancers and might predict the response to tamoxifen treatment **(A)** Representative immunostains for BCL9-2 on human breast tissue microarrays. Shown are examples for normal human breast tissues and breast cancers (CA) with (“+”) or without (“-“) positivity for ER or Her2 based on the immunoreactive scores. **(B)** Immunofluorescence stains and merged pictures for BCL9-2, ER and SMA. Shown are serial sections from a human breast tissue microarray with an example of normal human breast tissue and an ER+ breast cancer. The scale bar represents 50 μM. **(C, D)** Box plot analysis of the BCL9-2 immunoreactive score in normal human breast tissues (N; n=30) and breast cancer samples (CA; n=194). BCL9-2 scores were further plotted for the pathological grade (B; G1-G3: n= 16; 116; 38; respectively) and for ER/PR or Her2 positivity (C; triple negative: n=48; Her2+: n=64; ER/PR+: n=26; ER/PR and Her2+: n=53). P-values are indicated in the graphs with n.s. = not significant; *P<.05; ** *P*<.005; *** *P*<.001 (Mann-Whitney test). **(E)** Kaplan-Meier analysis for the relapse free survival of tamoxifen treated patients with ER+ breast cancers. BCL9-2 expression data were derived from microarray analyses [GSE 6532, 28]. High (n=129) or low (n= 134) BCL9-2 was relative to the median expression of all samples. Significance was calculated using the Cox Proportional Hazard Model.

**Figure 2:**
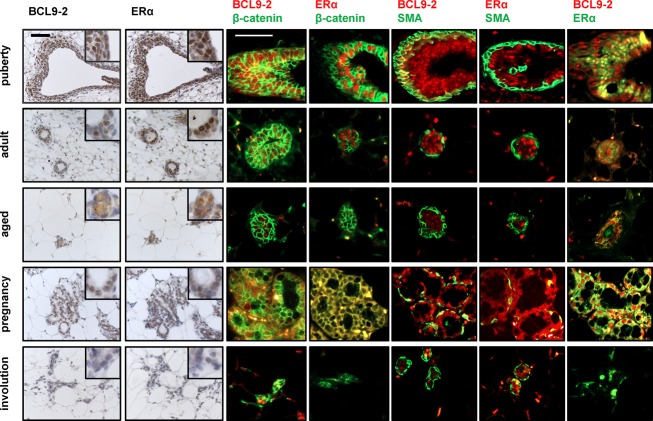
BCL9-2 is highly expressed during puberty and pregnancy in the normal mammary gland, but downregulated in the involuted breast BCL9-2 and ER immunostains on serial sections (left panel) and co-immunofluorescence staining for BCL9-2, ER, ß-catenin and SMA (right panel) in the mammary gland at different stages of postnatal development. Tissues from wild-type C57BL/6 mice at the age of 4 weeks (puberty), 4 month (adult virgin), 18 month (aged), during late pregnancy (E18.5) and on day 20 of involution after pregnancy were examined. Shown are for each stage representative examples of terminal end buds or alveoli. The scale bar represents 50 μM; inserts show the stains at higher magnification.

Since BCL9 proteins are implicated in cancer development and progression, we analyzed their expression and role in normal and malignant breast tissues. First, we characterized BCL9 proteins in human tissue samples and during different stages of postnatal mammary gland development in the mouse (Fig. 1 and 2, Suppl. Fig. 1). For immunostains we used our specific antibodies against BCL9 and BCL9-2 [15] and re-confirmed their specificity on mammary tissues by peptide competition (Suppl. Fig. 1D-F).

We performed immunohistochemistry and immunofluorescence analyses on commercially available tissue microarrays containing matched samples of adjacent normal and breast cancer samples (Fig. 1A, B and Suppl. Fig. 1A-C). We found that BCL9 was strongly expressed in epithelial cells of normal mammary tissues and was equally high in breast cancers (Suppl. Fig. 1A, B). In contrast, we detected only weak expression of BCL9-2 in normal breast tissues, whereas it was highly expressed in tumors, especially in ER+ cancers (Fig. 1A). We additionally performed co-immunofluorescence studies for BCL9-2 and ER to analyze their co-localization and for SMA (smooth muscle actin) that is expressed in myofibroblasts of the mammary gland (Fig. 1B and Suppl. Fig. 1C). In the adjacent normal breast, BCL9-2 was not detectable and nuclear ER was limited to single luminal cells that were surrounded by SMA positive myofibroblasts. In contrast, BCL9-2 was strongly expressed in the nuclei of breast tumor cells that also co-expressed nuclear ER and were negative for SMA (Fig. 1B and Suppl. Fig. 1C). These data confirm that BCL9-2 and ER co-localize in the nuclei of breast cancer cells.

Next we scored the immunohistochemistry staining intensity and the percentage of positive cells for BCL9-2 in a larger series of human breast cancer cases on tissue microarrays as previously described [15] (Fig. 1C, D). Scoring of the stains revealed that BCL9-2 was indeed significantly increased in all cancers cases compared to normal breast tissues (Fig. 1C; *P=0.001*), with highest expression in well differentiated tumors (*P=0.005, 0.002* and *0.046* for G1, G2 and G3, respectively). Moreover, we scored different cancer subtypes based on their ER, PR, and Her2 expression (see Suppl. Material and Methods) and confirmed highest levels of BCL9-2 in ER+ cancers. In detail, BCL9-2 was not elevated in triple negative cancers (*P=0.22)*, but in Her2+ tumors (*P=0.005)*. ER/PR positive cancers, with and without Her2 expression, showed highly significant upregulated BCL9-2 (*P=0.002* and *P<0.0001*, respectively; Fig. 1A, D).

We also assessed the clinical significance of BCL9-2 for the survival of ER+ breast cancer patients (Fig. 1E). For this, we re-evaluated microarray data from over 250 patients with early stage breast cancer and proven positive ER status [GSE 6532, 28]. We correlated BCL9-2 expression with the clinical outcome of the patients. First we separated high BCL9-2 (n=129 cases) versus low BCL9-2 (n=134) based on the median gene expression of all tumor samples. In fact, BCL9-2 correlated again with the pathological grade of the tumors and the overall survival (not shown). Remarkably, tamoxifen treated patients with high BCL9-2 in the tumors showed a significantly better survival than patients with low BCL9-2 (*P<0.0045*; Fig. 1E). In summary, BCL9-2 is highly expressed in ER+ human breast cancers and might predict the response to tamoxifen treatment.

### BCL9-2 is highly expressed in the normal mammary gland during puberty and pregnancy, but downregulated in the involuted breast

Next, we characterized the expression of BCL9 proteins during different stages of postnatal mammary gland development in the mouse (Fig. 2 and Suppl. Fig. 1). We stained mammary tissues of pubertal, virgin, pregnant and aged wild-type mice. BCL9 was strongly expressed in mammary epithelial cells at all ages (Suppl. Fig. 1D-F). In contrast, we detected strong BCL9-2 expression during puberty and in the pregnant mammary gland, while it was low in the involuted breast of aged animals and after pregnancy (Fig. 2). We asked if the BCL9-2 expression pattern correlates with ER expression in the postnatal mammary gland. For this we stained sequential tissue sections for BCL9-2 and ER (Fig. 2, left panel) and performed co-immunofluorescence stains of BCL9-2, ER, ß-catenin and SMA (Fig. 2, right panel). The terminal end buds of the outgrowing ducts in the pubertal mammary gland strongly expressed both, BCL9-2 and nuclear ER. In contrast, BCL9-2 was downregulated in the adult mammary gland and finally limited to single cells in the mature, involuted mammary epithelia of aged animals. Nuclear ER also dropped during postnatal development and was limited to few single mammary cells in aged mice. Highest BCL9-2 was again detected in late pregnancy, concomitant with strong cytoplasmatic and nuclear ER expression. During postlactational involution of the breast, BCL9-2 and ER were only weakly detectable in the remaining collapsed alveoli. After completion of the postlactational involution, BCL9-2 was again similarly low as in the virgin adult ductal epithelium. Thus, BCL9-2 and ER are highly expressed in the normal mammary epithelium during puberty and pregnancy. In contrast, BCL9-2 expression is almost completely lost in the postlactational and age-related involuted mammary gland.

### Overexpression of BCL9-2 *in vivo* delays the mammary involution and induces premalignant changes in transgenic mice

**Figure 3:**
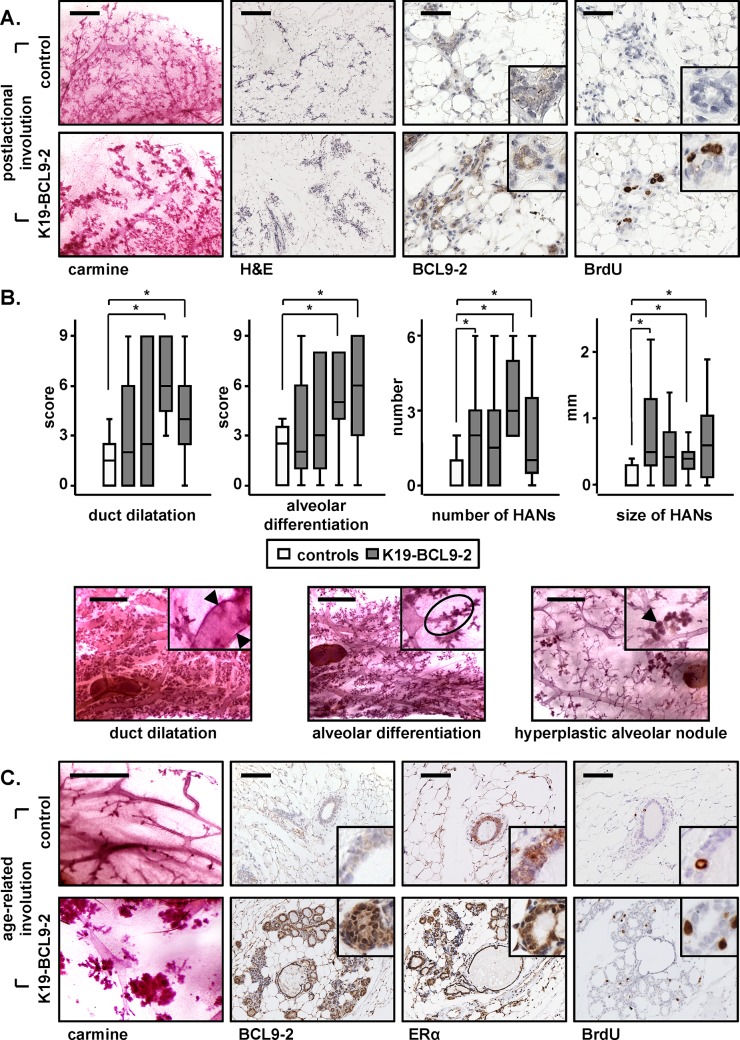
*In vivo* overexpression of BCL9-2 delays the postlactational and age-related involution and induces preneoplastic changes of the mammary gland in mice **(A)** Carmine stains and immunohistochemistry with the indicated antibodies of representative mammary glands of four month old non-transgenic and BCL9-2 females after pregnancy. Shown are mammary tissues on day 10 of involution. **(B)** Box-Plot analyses of the scoring (upper panel) and representative carmine stains (lower panel) for the indicated preneoplastic changes in the aged mammary gland. Age-matched non-transgenic (white bars) and BCL9-2 virgin females from four different founder lines (grey bars) were analyzed at 22.0 ± 2.0 month of age. Each group represents at least six animals. The asterisk marks significant differences with *P*<.05. **(C)** Representative stains of mammary glands from age-matched, 20 month old non-transgenic and BCL9-2 virgin females. Scale bars in the pictures represent 2 mm for carmine stains, 200 μm for H&E, and 50 μm for IHC. Inserts show the staining at higher magnification.

To study a potential role of BCL9-2 as proto-oncogene in the mammary gland, we analyzed the *in vivo* overexpression of BCL9-2 in transgenic mice. Our *K19-BCL9-2* mouse model (on a pure C57BL/6 background) induces BCL9-2 overexpression by the Keratin 19 (*K19*) gene promoter, which targets in the breast fully developed luminal cells and putative luminal progenitors [15; 29; 30]. We asked if BCL9-2 overexpression may affect the normal postnatal mammary development or can induce atypical preneoplastic lesions *in vivo*. For this, we analyzed BCL9-2 mice and age-matched non-transgenic controls by immunohistochemistry on tissue sections and carmine whole mount stains (Fig. 3).

First, we studied the postlactational involution in young animals after pregnancy (Fig. 3A). Remarkably, the remodeling of the breast to the prepregnant state was delayed in BCL9-2 transgenic females. The mammary glands retained large alveoli with high BCL9-2 that were actively proliferating as detected by BrdU stains. At this stage, alveoli of non-transgenic controls were collapsed, did not proliferate and BCL9-2 was low (Fig. 3A).

**Figure 4:**
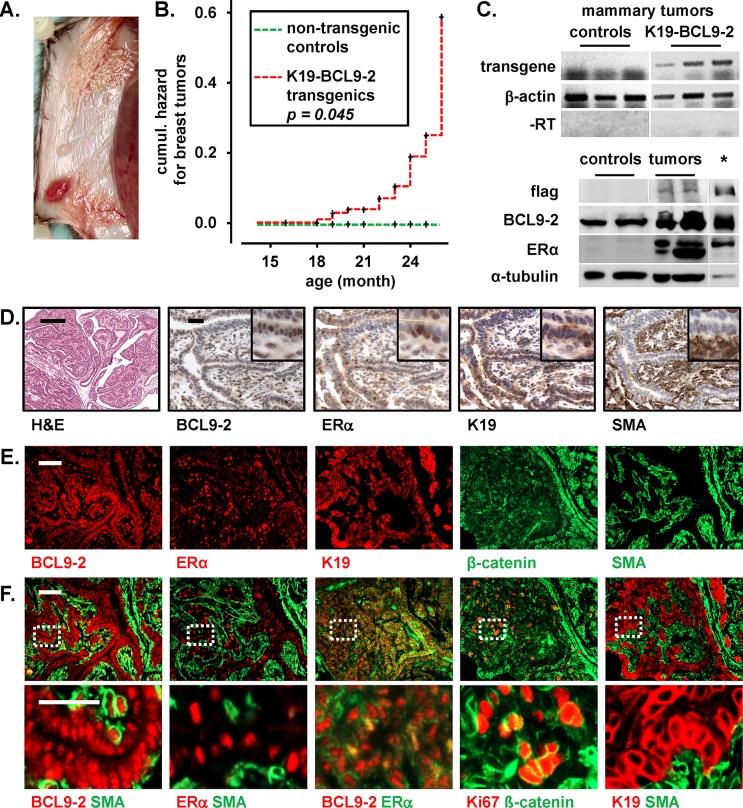
Aged BCL9-2 transgenic mice develop ER+ breast cancers **(A)** Representative macroscopic view of a mammary tumor from *K19-BCL9-2* mice in the inguinal mammary gland. The tumors were well vascularized. Note the concomitant enlarged ducts in the thoracic mammary gland. **(B)** Kaplan-Meier analysis of the cumulative hazard for the development of breast cancers in aged transgenic females (n=109 from six different founder lines). Note that non-transgenic controls (n=34) did not produce breast tumors (*P* log Rank=0.045; see also Suppl. Table 1 for tumor frequencies). **(C)** RNA and protein expression in mammary tumors from *K19-BCL9-2* transgenics. Upper panels: Transgene RNA expression in mammary tumors from *K19-BCL9-2* transgenics and tumors of APC^Min/+^ mice as controls. Reverse transcribed RNA and samples without prior reverse transcription (-RT) were analyzed by PCR with transgene specific and ß-actin primers. Lower panels: Western Blot analyses of primary cells from *K19-BCL9-2* mammary tumors and non-transgenic control breast tissues. Lysates were analyzed for the expression of flag-tagged transgenic and endogenous BCL9-2 (60μg) and ER (25μg) proteins. Loading was controlled by α-tubulin. Overexpressed proteins from transiently transfected HEK293 cells were used as positive controls (indicated by an asterisk). **(D)** Histopathology of a representative example for the ductal-like breast tumors from BCL9-2 transgenic female mice. Tissue sections were stained by H&E and with the indicated antibodies for cell specific markers. (E, F) Immunofluorescence **(E)** and co-immunofluorescence **(F)** stains for the indicated markers of epithelial cells and fibroblasts in an example of the ductal-like BCL9-2 tumors. Scale bars in the figures represent 200 μm (H&E) and 50 μm for IHC or IF. Inserts show the staining at higher magnification.

We also analyzed mammary tissues from aged control and BCL9-2 transgenic mice. Breast tissues of BCL9-2 overexpressing animals frequently showed macroscopically hyperplastic mammary glands with enlarged ducts, which was confirmed by carmine stains and on tissue sections (Fig. 3B, C and Fig. 4A). We scored the changes of mammary epithelium detected by carmine stains (see Suppl. Material and Methods) and found that the alveolar differentiation persisted and that the ducts were strongly dilated in aged BCL9-2 animals. At this stage, age-matched controls had almost completely lost all alveolar structures and the gland contained only large, undilated ducts (Fig. 3B, C). Moreover, BCL9-2 mice developed significantly more and larger hyperplastic alveolar nodules (HANs). Tissue sections of the transgenic glands were strongly positive for BCL9-2 and nuclear ER within the hyperplastic alveolar epithelium (Fig. 3C). Moreover, the alveolar foci were actively proliferating as indicated by BrdU stains. In contrast, the BCL9-2, ER and BrdU stains were restricted to single epithelial cells within larger ducts in the involuted mammary glands of aged control females (Fig. 3C). In summary, BCL9-2 overexpression induces premalignant changes of the breast and delays the normal postlactational and age-related mammary involution. Thus, sustained BCL9-2 expression *in vivo* might confer an increased risk for breast tumor development by inducing atypical preneoplastic lesions.

### Transgenic BCL9-2 overexpression induces ER+ mammary tumors in aged mice

We further monitored BCL9-2 mutant mice up to two years of age. Age-matched, non-transgenic littermates or C57BL/6 controls did not develop breast tumors. In contrast, transgenic females developed rapidly growing, macroscopic mammary tumors starting at 15 month of age (Fig. 4A). Tumor risk was significantly increased with age and tumors developed with similar frequencies in different founder lines (Fig. 4B and Suppl. Table 1). Of note, the tumor incidence of parous transgenic females was also significantly higher than of virgins (26 vs. 16%, Suppl. Table 1). Expression of the BCL9-2 transgene in the tumors was confirmed by RT-PCR using transgene specific primers and by Western Blots of tumor cell lysates using an antibody directed against the tagged transgene (Fig. 4C). Moreover, BCL9-2 and ER protein levels were strongly increased in the tumor cells compared to mammary control tissues as detected by Western Blots (Fig. 4C).

The majority of BCL9-2 breast tumors were histopathologically evaluated as ductal-like mammary tumors with transition to carcinomas (13 of 20 tumors, Fig. 4D-F and Suppl. Fig. 2A, B). Moreover, four tumors showed the morphology of lobular breast cancers as found in humans and the remaining cases were poorly differentiated tumors of the mammary gland (Suppl. Fig. 2C).

We further characterized the histopathology of BCL9-2 tumors by immunohistochemistry and co-immunofluorescence analysis (Fig. 4D-F and Suppl. Fig. 2A-C). We stained the tumors for the expression of BCL9-2 and hormone receptors. In addition, we performed staining for the epithelial cell markers Cytokeratin 19 (K19), ß-catenin and E-cadherin. Fibroblasts were stained with SMA and p63. Importantly, all ductal-like BCL9-2 tumors were highly positive for both BCL9-2 and nuclear ER, which was confirmed by co-immunofluorescence staining (Fig. 4 E, F). All ductal-like cancers strongly expressed K19 and showed membrane bound ß-catenin and E-cadherin (Fig. 4D-F and Suppl. Fig. 2A, B). Membranous Her2 was not detectable, indicating that the tumors mimic the luminal subtype of breast cancer. SMA expressing cells were negative for K19, while p63 stained the same SMA positive cells in the tumors (Fig. 4F and Suppl. Fig. 2C). Moreover, areas with BCL9-2, ER positive tumor cells where surrounded by a compartment with SMA expressing cells (Fig. 4E-F). Similarly, we found for the majority of breast cancer samples on the human tissue array the same staining pattern (Fig. 1B). Both human and mouse breast cancer tissues demonstrated that the BCL9-2+, ER+ tumor cell clusters were surrounded by SMA positive cells, suggesting to represent a stromal tumor compartment. All mouse BCL9-2 tumors were highly proliferative as detected by Ki67 and BrdU staining (Fig. 4F and Suppl. Fig. 2A).

The lobular-like BCL9-2 cancers were composed of highly proliferative, monomorphic tumor cells that had lost all epithelial markers including the expression of E-cadherin (Suppl. Fig. 2C). BCL9-2 and luminal epithelial markers were restricted to the few remaining ductal-like structures and to single cells within the tumors. However, lobular-like cancers also expressed high nuclear ER (Suppl. Fig. 2C).

In summary, *in vivo* overexpression of BCL9-2 induces the development of mammary tumors with high nuclear ER reminiscent of human ductal-like, ER and BCL9-2 positive breast cancers.

### Primary BCL9-2 mammary tumor cells respond to estrogen and tamoxifen treatment

**Figure 5:**
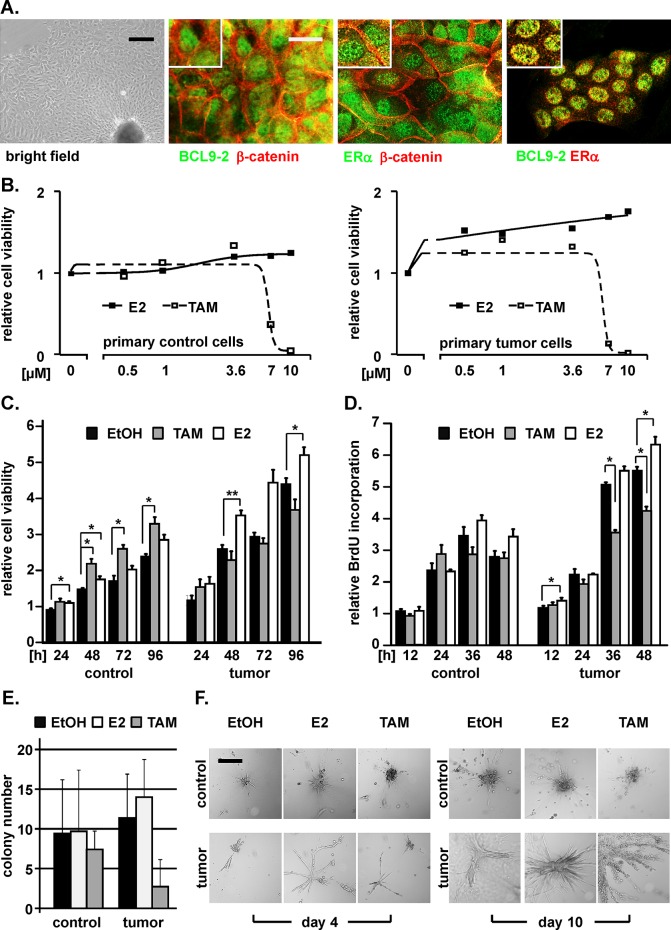
Primary breast tumor cells from BCL9-2 transgenic mice respond to estrogen and tamoxifen **(A)** Bright field image and co-immunofluorescence stains of primary cells established from mammary tumors of BCL9-2 females. Merged pictures of the co-stains with the indicated antibodies are shown. **(B)** Dose-response analysis of primary control and of primary BCL9-2 tumor cells for increasing concentrations of estrogen (E2) and tamoxifen (TAM), relative to vehicle treated cells. Cells were stimulated with the indicated concentrations for 48 hours and cell viability was determined by MTT assays. **(C-F)** Proliferation and colony formation of primary BCL9-2 tumor and non-transgenic mammary control cells. Cells were stimulated with 3.6 μM E2 or TAM and compared to vehicle (EtOH). (C, D) Proliferation at the indicated time points was determined by (C) MTT assays and (D) BrdU incorporation. (E) Colony formation of primary BCL9-2 tumor cells and of non-transgenic control cells cultured on collagen. Shown are the absolute colony numbers of untreated versus E2 and TAM treated primary cells after four days in culture. (F) Representative bright-field images of colonies from primary control and tumor cells treated with the indicated conditions, on day 4 and 10 after culture on collagen. The graphs show the mean of at least three independent experiments and of their standard error, relative to vehicle treated controls. * indicates significant differences for *P*<.05. Scale bars: 100 μm for bright field in (A) and 200 μm in (F).

To assess the growth potential and hormone sensitivity of the BCL9-2 tumors we established primary cell cultures of BCL9-2 breast tumors and of mammary glands from age matched, non-transgenic controls (Fig. 5 and Suppl. Fig. 3). We confirmed by immunofluorescence stains that the primary tumor cells retained all linage specific markers as detected by immunostains on tumor tissue sections (Fig. 5A and Suppl. Fig. 3A). The culture also contained single SMA positive cells. Importantly, primary tumor cells were highly positive for BCL9-2 and ER, which co-localized in the nuclei of primary tumor cells (Fig. 5A).

Next, we analyzed the proliferation capacity of the primary cells and their response to estrogen and tamoxifen treatment. Of note, mouse primary tumor cells stopped proliferating upon complete serum starvation, indicating that they require growth factors and hormones. Therefore we kept the cells in the supplemented media as previously reported for other primary cultures [31] and treated them with increasing concentrations of estrogen and tamoxifen (Fig. 5B-F). The proliferation of primary tumor cells was slightly stimulated in a dose- and time-dependent manner by estrogen as analyzed by cell viability and BrdU incorporation assays (Fig. 5B-D). The limited effects of estrogen might be due to the fact that our primary cells did not tolerate hormone starvation. We also analyzed the effects of increasing tamoxifen concentrations on cell viability and determined a non-toxic concentration for treatment of the primary cells (Fig. 5B, C). Importantly, tamoxifen treatment of the primary tumor cells led to a significant reduction of tumor cell growth even in fully supplemented culture media within 48 hours as determined by BrdU incorporation, while control primary cells did not respond (Fig. 5D).

We next analyzed colony formation and differentiation capacity of primary cells cultured on a collagen matrix (Fig. 5E, F). Primary control cells formed only small colonies within 4 days that did not proliferate or respond to estrogen and tamoxifen treatment. In contrast, primary tumor cells developed large colonies, which also slightly responded to estrogen treatment with increased colony numbers and larger colonies after 10 days. Remarkably, tamoxifen treatment strongly reduced colony formation of primary tumor cells within four days (Fig. 5E). In fact, tamoxifen led to a complete disintegration of the tumor cell colonies after ten days (Fig. 5F). Taken together, our data indicate that the growth of primary BCL9-2 mammary tumor cells is inhibited by tamoxifen treatment.

### BCL9-2 regulates ER signaling in human breast cancer cells independently of ß-catenin

**Figure 6:**
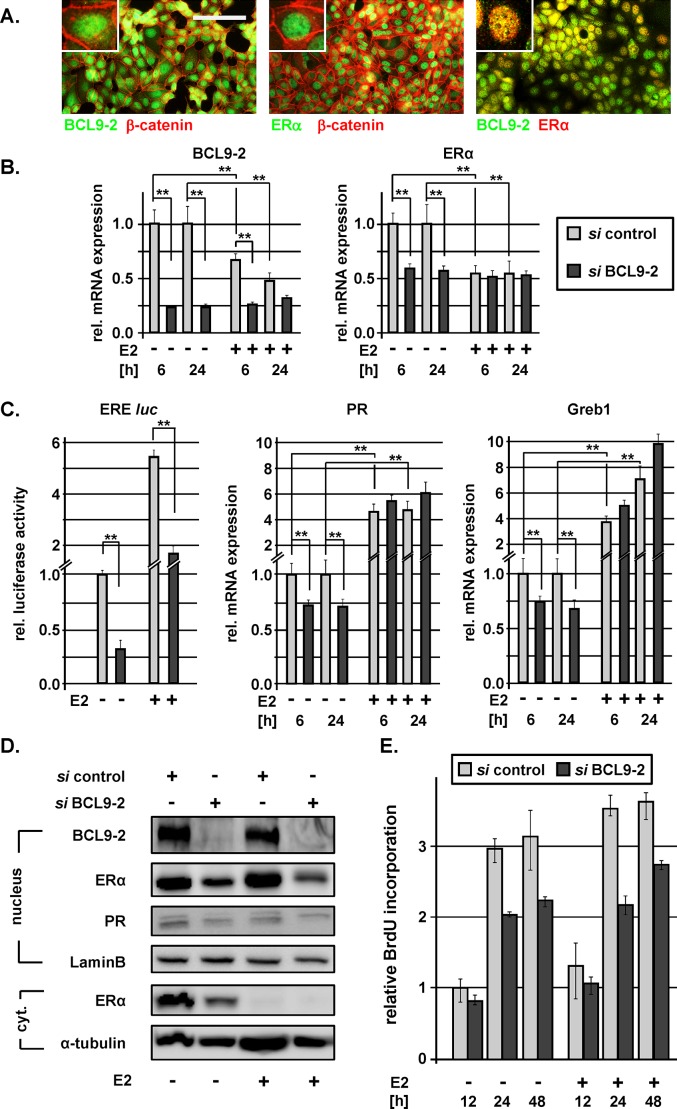
BCL9-2 regulates ER expression and modulates ER signaling in human breast cancer cells **(A)** Co-immunofluorescence stains for BCL9-2, ER and ß-catenin in MCF7 cells. The scale bar represents 50 μm. **(B-D)** The effects of BCL9-2 knockdown on ER expression and signaling in ER+ breast cancer cells. In all experiments, cells were pretreated with the indicated siRNAs for 48 hours. Cells were hormone-starved overnight, followed by treatment with 10 nM estrogen (+E2) or vehicle alone (-E2) for additional 6 or 24 hours. Graphs show the mean of at least three independent experiments and of their standard error, relative to control siRNA-treated cells. Significant differences are indicated with * for *P*<.05 and ** for *P*<.01. **(B)** qRT-PCR analysis of the RNA expression for BCL9-2 and ER after siRNA treatment against BCL9-2 in MCF7 cells followed by treatment with or without estrogen for 6 and 24 hours. **(C)** Luciferase activity of a reporter containing optimal ER responsive elements (ERE-luc) and qRT-PCR analysis of the indicated ER target genes in MCF7 cells after downregulation of BCL9-2 followed by treatment with or without estrogen for 6 or 24 hours. **(D)** Representative Western Blots of nuclear and cytoplasmatic (cyt.) fractions after BCL9-2 knockdown and 24 hours of estrogen treatment in MCF7 cells. Lysates were probed with the indicated antibodies. As loading controls, blots were re-probed with Lamin B and alpha-tubulin antibodies. **(E)** The effect of BCL9-2 knockdown on proliferation of MCF7 cells was determined by BrdU incorporation assays. Shown is a representative time course experiment performed in triplicates and the range for each time point. BrdU incorporation was calculated relative to control cells at 12 hours. Cells were pretreated with the indicated siRNAs for 48 hours, hormone starved overnight and incubated with and without 1μM E2 for the indicated time points.

Next, we studied the biological function of BCL9-2 in human breast cancer cells. First, we determined the expression of endogenous BCL9 and Pygo proteins by Western Blot and immunofluorescence analysis (Fig. 6A; Suppl. Fig. 4A and 5A). BCL9 was found at approximately equal levels in all breast cancer cell lines. In contrast, highest BCL9-2 was detected in ER+ MCF7 and T47D breast cancer cells and in Her2 positive SK-BR-3 cells. In addition, all three cell lines showed also high Pygo2 expression. Triple negative breast cancer cells and the fibroblastic MCF10a cells expressed much lower levels of BCL9-2 (Suppl. Fig. 4A), confirming our data from human tissue microarrays (see above). Immunofluorescence analysis revealed that BCL9-2 and ER co-localized in the nuclei of MCF7 and T47D cells. In contrast, ß-catenin was located at the cell membrane, but not in the nucleus together with BCL9-2 or ER in both cell lines (Fig. 6A and Suppl. Fig. 5A).

We then characterized the role of BCL9-2 in ER+ breast cancer cells by downregulation of the protein and compared the effects following knockdown of ß-catenin. We used the combination of two specific siRNAs against each transcript for RNA interference, which we found to be most effective for downregulation of the respective mRNA and protein at low concentration (Suppl. Fig. 4B, C).

First, we assessed the contribution of BCL9-2 to Wnt/ß-catenin signaling in these breast cancer cells. The transcriptional activity of the TOP/FOP Wnt-reporter was very low in MCF7 and almost not detectable in T47D cells. Knockdown or overexpression of BCL9-2 or ß-catenin had no significant effect on the Wnt-reporter activity, even after Wnt3a stimulation (Suppl. Fig. 6 A, B). Even though TOP/FOP reporter activity might not fully reveal canonical Wnt signaling activity, these data suggest that BCL9-2 does not contribute as co-activator for Wnt/ß-catenin signaling in ER+ breast cancer cells.

We further investigated the biological effects of BCL9-2 in MCF7 and T47D cells (Fig. 6B-E and Suppl. Fig. 5B-C). For this, we used hormone starved cells and thereafter re-stimulated with estrogen for 6 and 24 hours. First, we asked, if BCL9-2 might regulate ER expression. Remarkably, BCL9-2 knockdown significantly reduced ER RNA levels in hormone starved cells (Fig. 6B and Suppl. Fig. 5B). Following estrogen stimulation, RNA expression of ER was reduced as previously reported [32]. Interestingly, BCL9-2 RNA expression was also inhibited by estrogen treatment, suggesting a regulatory feedback loop also for BCL9-2 expression (Fig. 6B and Suppl. Fig. 5B). On the protein level, ER was reduced 72 hours after knockdown of BCL9-2, both in the nuclear and cytoplasmatic fraction of hormone starved cells (Fig. 6D). Data from at least three independent western blot analyses were quantified as shown in Suppl. Fig. 4D. Estrogen stimulation led to a complete translocation of the cytoplasmatic ER pool resulting in increased protein levels in the nuclei. However, nuclear ER protein was reduced in estrogen stimulated cells following knockdown of BCL9-2 compared to control siRNA treated cells (Fig. 6D, Suppl. Fig. 4D).

Next, we asked if BCL9-2 also affects ER signaling. Knockdown of BCL9-2 strongly reduced the activity of an ER responsive (ERE) luciferase reporter in MCF7 and T47D cells, both in hormone starved cells and after stimulation with estrogen (Fig. 6C and Suppl. Fig 5C). Moreover, we analyzed the expression of the ER target genes progesterone receptor (PR) and GREB1 (growth regulation by estrogen in breast cancer 1) and asked if downregulation of ER protein following BCL9-2 knockdown also affects ER target gene expression. In fact, the RNA levels of PR and GREB1 were significantly reduced in hormone starved cells when we down-regulated BCL9-2 (Fig. 6C). PR protein was also reduced 72 hours after BCL9-2 downregulation (Fig. 6D and Suppl. Fig. 4D). Estrogen treatment induced the expected upregulation of ER target gene transcription, which did override the effects of BCL9-2 on ER expression. This may reflect that the remaining ER protein after BCL9-2 knockdown is still sufficient to activate the transcription of ER target genes following estrogen stimulation and thus rescues the effects of BCL9-2 knockdown.

In addition, we analyzed if BCL9-2 also affects cell growth. Knockdown of BCL9-2 strongly reduced the proliferation of hormone starved and estrogen stimulated cells as determined by BrdU incorporation (Fig. 6E). Of note, the reduced cell proliferation in BCL9-2 depleted cells was not compensated by high doses of estrogen (Fig. 6E). In contrast, knockdown of ß-catenin in MCF7 cells affected neither the ERE reporter activity nor the expression of ER or the target genes. Furthermore, knockdown of ß-catenin did not inhibit cell proliferation (Suppl. Fig. 6C-F). In summary, BCL9-2, but not ß-catenin, is important for the expression of ER and regulates the proliferation of human ER+ breast cancer cells.

### BCL9-2 regulates ER transcription by interaction with Sp1 through the proximal ESR1 gene promoter

Our data suggested that BCL9-2 modulates ER expression on the transcriptional level. To further address the underlying molecular mechanism, we analyzed the recruitment of BCL9-2 to the human ESR1 gene promoter by chromatin-immunoprecipitation (ChIP) and performed co-immunoprecipitation studies in ER+ breast cancer cells.

**Figure 7:**
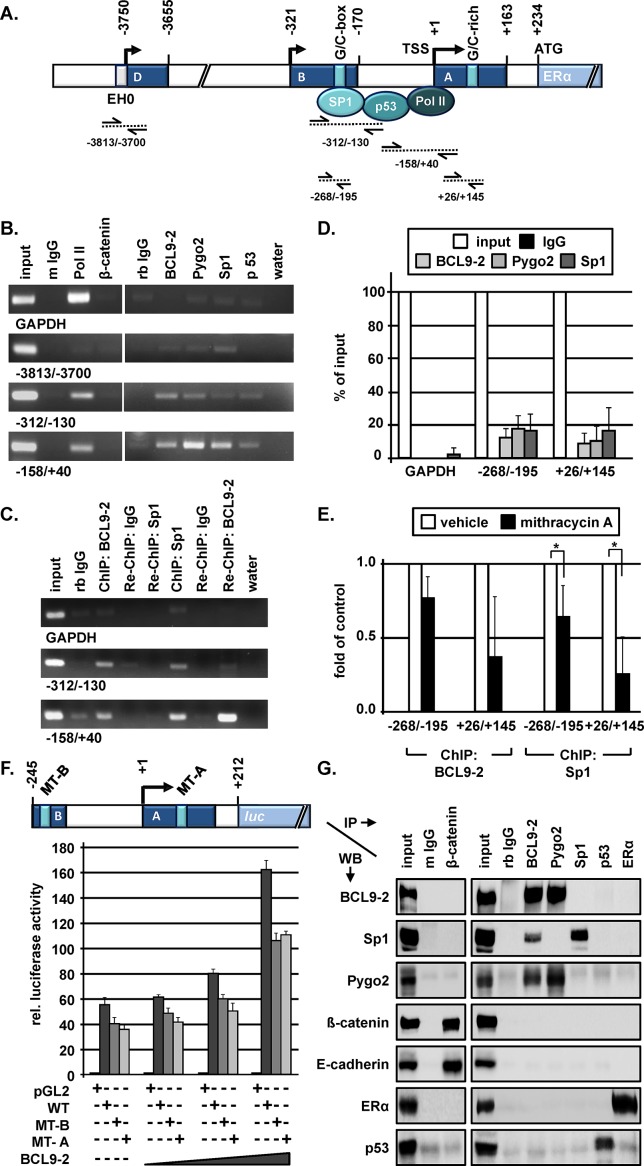
BCL9-2 regulates the transcription of ER in the proximal promoter and interacts with Sp1 in human breast cancer cells **(A)** Schematic view of regulatory elements within the upstream sequence of the human ESR1 gene that are important for ESR1 gene transcription in breast cancer cells (according to Kos et al., 2001). One previously described G/C-box in promoter B and a G/C-rich element in promoter A as potential Sp1 binding sites are also indicated. The amplified promoter regions by conventional PCR and qRT-PCR following ChIP are indicated below the scheme. **(B)** Representative examples of the PCR analysis for the indicated promoter fragments. ChIP experiments were performed for the indicated promoter regions of the ESR-1 gene and the GAPDH promoter as control. **(C)** Re-ChIP experiments of BCL9-2 and Sp1 for the two most proximal promoter regions of the ESR1 gene in MCF7. Representative examples of the PCR analysis following ChIP and re-ChIP with the indicated antibodies and IgG controls are shown. Rabbit IgG (rb IgG) was used as negative control. **(D)** qRT-PCR analyses for the indicated promoter regions of at least three independent ChIP experiments in MCF7 following immunoprecipitation of the DNA with the indicated antibodies. We performed the absolute quantification using the regression analysis method and calculated the values as percent of input DNA after normalization to the control IgG. **(E)** qRT-PCR of at least three ChIP experiments following mithramycin A treatment. MCF7 cells were treated with 10 nM mithramycin A or vehicle (0.1% EtOH) for 24 hours prior to fixation. The fold enrichment was calculated using the regression analysis method and normalized to control IgG. **(F)** Luciferase activity of a reporter containing the most proximal ESR1 gene promoter (WT = wild type), of a G/C box mutant in promoter B (MT-B) and of a G/C-rich element in promoter A (MT-A) as indicated in the scheme. The graph shows a representative dose response experiment performed in triplicates and their range. HEK293 cells were transfected with 100 ng reporter constructs and 0, 12.5, 50 und 100 ng BCL9-2 expression constructs for 72 hours. **(G)** Western Blots (WB) analyses of MCF7 cell lysates after co-immunoprecipitation (IP) with the indicated antibodies. As negative controls, mouse and rabbit IgG's were used.

Since BCL9-2 lacks a conserved DNA binding domain, we included transcription factors that were previously described to bind within the ESR1 gene promoter and activate ER transcription in breast cancer cells (Fig. 7A). First, we analyzed three larger regions in the upstream DNA sequence of the ESR1 gene by conventional PCR following ChIP. The first fragment covered the most proximal promoter A [based on the nomenclature by 33] that is occupied by a complex containing Sp1, p53 and other factors of the basal transcription machinery [7]. Although the binding sites for Sp1 in promoter A have not been described yet, our analyses using Transfac Patch tool [34] or JASPAR database [35] revealed that the ESR1 gene promoter A contains multiple G/C-rich sequences surrounding the transcription start site.

The second region included promoter B with a classical G/C box, which has been previously shown to bind Sp1 in breast cancer cells [5]. Moreover, we analyzed a region of the more upstream promoter D that contains an enhancer element (“EH0”) that is also activated in ER+ breast cancer cells [36]. We confirmed the occupancy of RNA polymerase II, Sp1 and p53 in the proximal promoter A (Fig. 7B). Remarkably, also BCL9-2 and Pygo2, but not ß-catenin, were clearly enriched at this region as demonstrated by repeated ChIP experiments in MCF7 cells. Moreover, BCL9-2 and Sp1 were also present at the promoter B that contains the G/C box, weakly together with Pygo2 and p53. In contrast, we did not detect an apparent enrichment of BCL9-2 or Pygo2 at the distal promoter D or the control GAPDH promoter (Fig. 7B). To further confirm the co-occupancy of BCL9-2 and Sp1 in the most proximal ESR1 gene promoter regions, we additionally performed re-ChIP experiments. Indeed, following ChIP with Sp1 we re-immunoprecipitated both promoter fragments with BCL9-2 (Fig. 7C). Vice versa, we also found Sp1 after BCL9-2 ChIP, however weaker possibly due to lower antibody efficacy for the immunoprecipitated complexes.

To further quantify the enrichment of BCL9-2, Pygo2 and Sp1, we designed specific primers for promoter B covering the known G/C Box and for sequences downstream of the transcription start site of promoter A that contained additional G/C rich elements (Fig. 7D, E). We performed qRT-PCR analyses following ChIP and found again the recruitment of BCL9-2 and Pygo2, together with Sp1, to promoter A and B of the ESR1 gene, but not to the GAPDH control (Fig. 7D). Moreover, we performed ChIP experiments after treatment of MCF7 cells with mithramycin A that inhibits Sp1 binding to the DNA [6]. We asked if reduced Sp1 binding also decreases the recruitment of BCL9-2 to the ESR1 gene promoter. In fact, within 24 hours of mithramycin A treatment, the recruitment of both Sp1 and BCL9-2 was reduced at both promoter fragments as determined by qRT-PCR (Fig. 7E). Thus, BCL9-2 regulates ER transcription through recruitment to DNA sites that are occupied by Sp1 in the most proximal promoters.

We further analyzed the contribution of BCL9-2 as co-regulator of ER transcription and performed luciferase reporter assays in HEK293 cells, which do not express endogenous BCL9-2, but Sp1 and low levels of ER (Suppl. Fig. 4A). For this, we generated a minimal luciferase reporter construct, which covers the most proximal promoter A and B of the ESR1 gene. Moreover, we mutated the previously described G/C box in promoter B and one additional G/C rich element in promoter A based on our sequence analyses (Fig. 7F). The basal activity of the wild type ESR1 reporter was slightly reduced by both mutations of the G/C rich elements in promoter A and B, respectively and not further reduced by a double mutant (Fig. 7F and data not shown). Remarkably, overexpression of increasing amounts of BCL9-2 increased the activity of the minimal wild type ESR1 reporter (Fig. 7F). BCL9-2 also activated the mutant ESR1 reporters. However, the activation for both mutants was lower compared to the wild type reporter when we titrated increasing amounts of BCL9-2 (Fig. 7F). These data indicate that BCL9-2 co-activates ER transcription, which relies on several G/C-rich elements representing potential Sp1 binding sites in the most proximal ESR1 gene promoters.

Finally, we analyzed the interaction of endogenous proteins by co-immunoprecipitation in MCF7 (Fig. 7G), T47D (Suppl. Fig. 5D) and mouse BCL9-2 breast cancer cells (Suppl. Fig. 3B). As expected, we found that BCL9-2 consistently co-precipitated with Pygo2 in all analyzed cancer cells. Remarkably, BCL9-2 also co-precipitated with Sp1. Vice versa Pygo2 co-precipitated with BCL9-2, but barely detectable with Sp1. ß-catenin co-precipitated with E-cadherin, but not with any other analyzed protein in the breast cancer cells. Interaction of p53 with Sp1 or BCL9-2 was not detectable by co-immunoprecipitation, which presumably requires stabilization of the protein as previously described [27]. Of note, immunoprecipitation with the Sp1 antibody did not co-precipitate BCL9-2 or ER, as previously described, which might be due to limited amounts or competition for binding of endogenous proteins. Of note, we also did not detect ER after precipitation of BCL9-2 or Pygo2, suggesting that they do not interact. The same results were found in T47D human breast cancer cells and in our primary BCL9-2 mouse tumor cells (Suppl. Fig. 3B and 5D). In summary, we have identified Sp1 as a novel binding partner of BCL9-2, which was previously unrecognized. This interaction might explain the ß-catenin independent functions of BCL9-2 to drive tumorigenesis. Our data uncover a novel mechanism for BCL9-2 as co-factor, which acts together with Sp1 and regulates ER transcription through the proximal ESR1 gene promoter in breast cancer cells.

## DISCUSSION

BCL9-2 belongs to the BCL9/Legless family that act together with Pygopus proteins as nuclear co-activators of canonical Wnt/ß-catenin signals in development and tumorigenesis [11; 12; 14; 15; 18]. Our previous work indicated that the BCL9-2 proto-oncogene harbors additional ß-catenin independent functions that promote tumorigenesis [15].

We show here, that BCL9-2 overexpression *in vivo* induced the formation ER+ breast tumors in aged transgenic mice. Females developed breast cancers, which exhibited a ductal-like morphology and highly expressed nuclear ER, similar to human ER+ breast cancers [1; 37]. We confirmed with specific markers, that the mouse tumors might represent a model for human ER+ breast cancers since we detected strong co-expression of nuclear ER and BCL9-2 both in human and mouse BCL9-2 tumor samples. Moreover, we found that human and mouse breast cancers contained a putative stromal compartment with SMA positive cells, surrounding the ER+, BCL9-2+ tumor cell clusters. Of note, SMA, a marker of myofibroblasts, is not only expressed in basal-like, hormone receptor negative cancers [38], but was also previously reported for human ductal ER+ breast cancers [39]. Moreover, SMA positive cells in breast cancers might represent activated myofibroblasts in the stroma and mark putative cancer-associated fibroblasts [40; 41]. Interestingly, cancer-associated fibroblasts might be the source of estrogen production in breast cancer [42]. Of note, estrogens are primarily produced outside of the ovary in postmenopausal women and in breast cancers [43], and similarly, in our mouse model aged females developed ER+ breast tumors. We also show that the proliferation of primary BCL9-2 tumor cells was sensitive to estrogen and tamoxifen treatment, suggesting a functional role of BCL9-2 to control proliferation in breast tumors. Correspondingly, human ER+ breast cancer patients with high BCL9-2 in the primary tumor showed a highly significant better overall survival when treated with tamoxifen (see below).

Our BCL9-2 transgenic mouse model represents one of the few genetic mouse models which develop ER+ breast tumors [8]. The phenotype of BCL9-2 mammary tumors shares several intriguing similarities with tumors from MMTV-Wnt1 transgenics in which we also found high BCL9-2 expression (Zatula and Brembeck, unpublished observations). It was suggested that activation of the Wnt1 proto-oncogene targets a common luminal progenitor, which results in ER+ mammary cancers [25; 44]. MMTV-Wnt1 mammary tumors are also at least in part dependent on ER signaling, since tumorigenesis was strongly delayed when crossed with ER knockout animals [45]. Conditional deletion of Pygo2 in MMTV-Wnt1 animals similarly delayed the tumor onset, indicating that Pygo2 also enhances Wnt1 driven tumorigenesis, remarkably without affecting the Wnt/ß-catenin signaling output [17]. Thus, BCL9-2 and its binding partner Pygo2 apparently drive breast tumor development *in vivo* by ß-catenin independent mechanisms. Further investigations are required to analyze the interplay of Wnt1/BCL9-2/Pygo2 for the induction of ER+ breast tumors *in vivo*.

However, Wnt1 is not expressed in the normal mammary gland and is not overexpressed in human breast cancer [46; 47]. In contrast, we show here that BCL9-2 was highly expressed in the normal breast during proliferative, hormone dependent stages with increased ER expression, i.e. during puberty and pregnancy [48]. Accordingly, BCL9-2 was lost upon the normal postlactational and age-related lobular involution, possibly as a consequence of the loss of hormonal stimuli [43]. BCL9-2 overexpression also conveys premalignant changes of the breast *in vivo* since we found more hyperplastic alveoli in aged BCL9-2 females. Such preneoplastic foci in mutant mice indeed harbor a malignant potential [43; 49]. Moreover, the postlactational and the age-related lobular involution of BCL9-2 females was delayed with persistence of proliferating alveoli, similar to the disturbed mammary involution of MMTV-Wnt1 and other mouse models [43]. In human, degradation of alveoli after pregnancy and in postmenopausal women was suggested to be protective against breast cancer [43]. Thus, persisting BCL9-2 expression disturbs the proper involution and may increase the cancer risk.

Our results from human breast cancer cells further argue for a role of BCL9-2 as regulator of ER expression in breast cancer. We found highest BCL9-2 in ER+ human breast cancer cells and show that BCL9-2 knockdown strongly regulated ER expression on the transcriptional level, which resulted in reduced ER protein level. ER target gene expression was also reduced following knockdown of BCL9-2, which was in part rescued by estrogen treatment possibly due to the nuclear accumulation of preexisting ER protein [32]. As biological consequences, depletion of BCL9-2 also reduced the growth of ER+ breast cancer cells, which may reflect impaired additional non-genomic functions of ER after BCL9-2 downregulation [1].

We have further addressed the contribution of the BCL9-2 co-factor for the transcriptional regulation of ESR1 gene expression in breast cancer cells. ER transcription is initiated from several promoters which reside within 15 kB upstream of the ESR1 gene locus [33]. Moreover, the promoters are differentially used in normal ER expressing tissues or in cancer cells [33; reviewed in 50]. The most proximal promoters are predominantly activated in human breast cancer cells, while a more distal promoter at -3 kB is alternatively utilized in normal mammary epithelia, but far less in breast cancer cells [5; 51]. The proximal promoters harbor several G/C rich elements as putative Sp1 binding sites and Sp1 has been shown to be an important regulator of ESR1 gene expression in breast cancer cells [5-7]. Sp1, a member of the Sp/Krüppel-like transcription factor family, is ubiquitously expressed in normal tissues, but highly overexpressed in ER+ breast cancers [4]. Previous studies have identified Sp1 binding to the most proximal ESR1 gene promoters, which recruits a multi-protein complex to initiate ER transcription [5-7]. It was shown that promoter B harbors a classical G/C box that is recognized by Sp1 [5; 6]. Moreover, we identified by promoter sequence analyses multiple G/C-rich sequences in the ESR1 gene promoter A. In fact, our ChIP analyses confirmed that Sp1 and BCL9-2 are recruited both to promoter A and B. Furthermore, using ESR1 reporter constructs, we found that BCL9-2 co-activates the reporter to a similar extent as previously demonstrated for Wnt-activity [11; 15; 52]. Mutation of the G/C-rich elements in the ESR1 reporter slightly reduced the basal activity. However, co-activation by BCL9-2 was impaired by mutations of the potential Sp1 binding sites in promoter A and B, although we did not identify the exact binding site. It is possible that additional suboptimal sites might be also recognized by Sp1 as previously reported [53; 54] and may contribute to the activation by Sp1 and BCL9-2. In summary, we provide evidence that BCL9-2 is recruited to the proximal ESR1 gene promoters together with Sp1 and co-activates basal ER transcription.

Moreover, we demonstrate by co-immunoprecipitation that BCL9-2 interacts with Sp1, which may explain the mechanism how BCL9-2 and Pygo2 are recruited to the ESR1 promoter. In fact, our ongoing work further indicates that the Sp1-BCL9-2 interaction drives the expression of additional target genes in cancer cells and that this interaction does not require the ß-catenin binding domain of BCL9-2 (Wiese and Brembeck, manuscript in preparation).

So far, transcriptional regulation of ER expression by components of canonical Wnt-signaling has not yet been described. A functional interaction between ER and ß-catenin was reported for Drosophila [55]. However, we did not detect an apparent interaction of endogenous ER and ß-catenin or BCL9-2 proteins. Moreover, aberrant activation of Wnt/ß-catenin signaling is not associated with luminal-like breast cancers [19]. Our data did not provide evidence for elevated canonical Wnt-signaling in ER+ human breast cancer cells, which was also previously reported [55]. In fact, we did not see regulation of ER expression after knockdown of ß-catenin in ER+ breast cancer cells. This is the first report that BCL9-2, previously characterized as co-activator of Wnt/ß-catenin signaling, regulates ER expression at the transcriptional level by interaction with Sp1 through the proximal ESR1 gene promoter, apparently independently of ß-catenin.

Our data also suggest a feedback loop between BCL9-2 and ER expression, since RNA levels of both, BCL9-2 and ER, were downregulated after estrogen stimulation. This is consistent with previous reports, that the expression of ER and its transcriptional activators are downregulated by estrogen [32; 56]. So far, transcriptional regulation of BCL9-2 has not yet been studied in detail, although we have previously shown that the BCL9 and Pygo genes are not Wnt/ß-catenin targets in cancer cells [15]. However, we identified several putative ERE elements in the upstream sequence of the BCL9-2 gene suggesting ER as a potential regulator of BCL9-2 transcription (Zatula and Brembeck, unpublished observations). Of note, the BCL9-2 gene resides on 11q, which is one of the most frequently rearranged chromosomal arms with repeated amplifications in human breast cancers [57]. In future, whole genome sequencing of cancer patient's samples will provide more information about possible rearrangements of the BCL9-2 locus. In summary, our findings indicate that the expression of BCL9-2 and ER are tightly connected in breast cancer cells.

Finally, our study revealed the potential clinical significance of BCL9-2 for human breast cancer. We demonstrate that BCL9-2, but not the BCL9 homolog, is overexpressed in most cancers and this was associated with the pathological grade. Most importantly, BCL9-2 expression correlated with hormone receptor positivity of the cancers: Triple negative breast cancers showed no BCL9-2 overexpression, but BCL9-2 was elevated in Her2 positive tumors, confirming a previous study [58]. Highest BCL9-2 was found in ER+ tumors, representing the luminal subtypes of breast cancers. Thus, high BCL9-2 is associated with ER+ human breast cancer, as we found in our animal model. From the clinical aspect, we propose that high BCL9-2 in the primary tumor might predict a favorable therapy response to anti-estrogen treatment, based on our data analysis of tamoxifen treated patients with ER+ breast cancers [28]. The primary response to tamoxifen depends indeed on the maintenance of ER expression [1; 28; 32; 56].

In summary, BCL9-2 acts as a novel molecular determinant of ER expression. This might provide a possible molecular mechanism how ER expression is maintained in breast cancers that overexpress BCL9-2 and thus are sensitive to tamoxifen treatment. For future clinical applications, assessing the levels of BCL9-2 in human breast tumors could be valuable to determine if a patient will benefit from anti-estrogen therapy.

## MATERIALS AND METHODS

### Animal Models

All animal experiments were performed in accordance with German guidelines and approved by governmental authorities. Generation of *K19-BCL9-2* mice was previously described [15] and animals were maintained on a pure C57BL/6 background. In all experiments, age-matched, non-transgenic littermates were used as controls.

### Immunohistochemistry, immunofluorescence and Whole Mount Carmine Stains

Immunohistochemistry and immunofluorescence were performed according to standard protocols; additional details are in Suppl. Material and Methods. Antibodies are listed in Suppl. Table 2. Inguinal mammary glands were fixed in 4% PFA and stained with carmine alum solution for 3 hours. Scoring of preneoplastic changes of the mammary glands was performed by multiplication of the scores for the severity and the amount of affected tissue (Suppl. Table 4).

### Primary cell culture, proliferation and collagen assays

Normal and tumor tissues were dissected from mammary glands of mice and transferred into isolation medium consisting of DMEM/F12 (Invitrogen) supplemented with 5% FBS, 20ng/ml mEGF, 5 μg/ml insulin,10 ng/ml dexamethasone, 10 mM nicotinamide (Sigma-Aldrich), 1×MEM non-essential amino acids (Invitrogen), 1× insulin-transferrin-selenium (Invitrogen), and 1% penicillin-streptomycin. Tissues were dissociated and digested in collagenase/hyaluronidase-solution (Stemcell Technologies, 1.5 hours at 37°C) followed by dispase digestion (1 U/ml, Sigma-Aldrich) for an additional hour. Tissues were filtered with a 40 μm cell strainer and further cultured with the supplemented isolation medium. Cell viability was determined by standard 3-(four, 5-dimethyl-2-thiazlyl)-two, 5-diphenyl-2H-tetrasolium bromide (MTT) assays. BrdU incorporation was measured using the colorimetric ELISA BrdU Kit according to manufacturer`s protocol (Roche). Cells were incubated with MTT or BrdU solution 3 or 12 hours prior to measurement, respectively. Additional details are in Suppl. Material and Methods.

### RNA Interference, Luciferase assays and qRT-PCR

MCF7 and T47D cells were obtained from ATCC and cultured in DMEM-GlutaMAX (Invitrogen) supplemented with 10% FCS and 1% penicillin-streptomycin. For hormone starvation, cells were cultured in phenol red-free DMEM (Biochrom) supplemented with 5% charcoal-dextran-treated FCS and 1% penicillin-streptomycin overnight followed by treatment with 17β-estradiol or vehicle (0.1% EtOH). For RNA interference, pools of two specific BCL9-2 or ß-catenin siRNAs and a pool of non-targeting control siRNA (Dharmacon-Thermo Fisher Scientific) were transfected at a final concentration of 25 nM for each single siRNA, using Lipofectamine2000 (Invitrogen). For ERE luciferase reporter assays, cells were pretransfected with siRNAs for 48 hours and re-transfected with 200 ng ERE-luciferase reporter and 25 ng tk-Renilla control for further 36 hours. The minimal ESR1 wild type and G/C box mutant promoter reporter were generated by PCR (primers are in Suppl. Table 3) and subcloned into pGL2 basic (Promega). Cells were transfected with 100 ng reporter and pcDNA-flag BCL9-2 expression constructs for 72 hours. In all luciferase reporter assays, firefly luciferase values were normalized to Renilla controls. RNA was isolated using Tri-Reagent (Ambion-Invitrogen) and reverse-transcribed using MMLV-RT (Thermo Fisher Scientific). qRT-PCR was performed with absolute SYBR green (Sigma-Aldrich) on the ABI Prism 7900HT (Applied Biosystems). Gene expression was calculated relative to actin as internal control. Primers are listed in Suppl. Table 3.

### Western Blot, co-immunoprecipitation (co-IP) and Chromatin immunoprecipitation (ChIP)

For Western Blot analysis, lysates were resolved by SDS-PAGE electrophoresis according to standard protocols. Quantification of protein levels after BCL9-2 knockdown was performed by densitometry using ImageJ software. Co-immunoprecipitation was performed on whole cell lysates using Protein A sepharose (GE Healthcare). ChIP and re-ChIP assays were performed after chromatin crosslink with 1% formaldehyde and sonification using the EZ-ChIP Kit (Merck Millipore) according to manufacturer`s protocol. The purified immunoprecipitated DNA was analyzed by standard PCR and qRT-PCR. Additional details are in Suppl. Material and Methods, antibodies and primers in Suppl. Tables 2 and 3.

### Human Tissue Array

Human breast cancer tissue arrays were purchased from Pantomics, Richmond, USA (BRC481 and BRC482), and US Biomax, Rockville, USA (BR1503). All arrays provided data for histology, TNM classification, pathological grade, and immunohistochemistry for ER, PR, and Her2. The arrays contained tissues from 30 patients with normal histology and 99 cancer patients, each tumor spotted in duplicates. Scoring of the immunostains is described in Suppl. Material and Methods.

## SUPPLEMENTAL MATERIAL, FIGURES AND TABLES


