# Supplementary material for: The BCL9-2 proto-oncogene governs estrogen receptor alpha expression in breast tumorigenesis
Source: Oncotarget. 2014 Jul 25;5(16):6770–87. doi: 10.18632/oncotarget.2252 (PMC4196162; doi:10.18632/oncotarget.2252)
Supplement: Supplementary file 1 [file oncotarget-05-6770-s001.pdf]

The BCL9-2 proto-oncogene governs Estrogen Receptor alpha expression in breast tumorigenesis

Supplemental Material

Immunohistochemistry and immunofluorescence

Prior to necropsy, mice were injected with 0.1 mg BrdU/g for two hours. Tissues were fixed in 4% paraformaldehyde. Antigen retrieval of serial sections was performed by heating in 10 mM Tris, 1 mM EDTA (pH 9.0). Antibodies used for immunohistochemistry and immunofluorescence are in Suppl. Table 2. Staining was detected with the streptavidin-peroxidase method (Dako) or Alexa488- (Molecular Probes), Cy2- or Cy3- (Jackson Immuno Research) conjugated secondary antibodies.

Cell proliferation and collagen assays of primary cells

For 2D-collagen assays, 96-well plates were coated with 50 µl collagen matrix (DMEM/F12 containing 1.5 mg/ml collagen solution (Sigma-Aldrich), 2.2 mg/ml $NaHCO_3$, 20 mM Hepes, 5% FCS). $1x10^3$ cells in 100 µl Opti-MEM were seeded overnight onto collagen coated wells. Cells were treated with 17β-estradiol or tamoxifen (Sigma-Aldrich), dissolved in ethanol at a final concentration of 0.1%, suspended in DMEM/F12 media containing 5% FCS, 20 ng/ml mEGF, 5 µg/ml insulin, and 1% penicillin-streptomycin.

Wnt Reporter assays

TOP/FOP reporter activity was determined following knockdown of BCL9-2 or β-catenin in MCF7 and T47D. Cells were pre-transfected with siRNAs for 48 hours followed by transfection with 100 ng TOP or FOP luciferase reporters and 25 ng tk-Renilla controls for additional 24 hours. For TOP/FOP reporter assays after overexpression of BCL9-2 or β-catenin, cells were transfected with 100 ng β-catenin or 200 ng BCL9-2 expression plasmids,

100 ng TOP or FOP reporter and 25 ng tk-Renilla control for 48 hours followed by treatment with Wnt3a- or control-conditioned media produced in L cells (ATCC) for additional 24 hours. Firefly luciferase values were normalized to Renilla controls.

Chromatin immunoprecipitation (ChIP), re-ChIP and Immunoprecipitation

ChIP and re-ChIP assays on MCF7 were performed after chromatin crosslink with 1% formaldehyde and sonification on a Diagenode BioRuptor (3-times 30 cycles of 10 sec on/off at high power) using the EZ-ChIP Kit (Merck Millipore) according to manufacturer`s protocol. For ChIP assays after mithramycin A treatment, MCF7 were pretreated 24 hours either with 0.1% EtOH as vehicle or 100 nM mithramycin A (Sigma). The elution of DNA and reverse protein/DNA crosslink was performed using Chelex-resin solution (Bio-Rad Laboratories) or using IPure kit (Diagenode) according to manufacturer`s protocol. Mouse and rabbit IgG (m IgG, rb IgG) were used as negative controls and for the normalization of the amplified signals. The fold enrichment was calculated as percent of input DNA. For co-IP's, cells were incubated on ice in RIPA buffer (50 mM Tris-HCl, pH 7.4; 150 mM NaCl; 1 mM EDTA, pH 8; 1% NP40; 0.25% Na-desoxycholate) containing 1x protease inhibitor cocktail (Roche). After centrifugation, the supernatants were incubated with the respective antibodies and Protein G sepharose (GE Healthcare) according to standard protocols. The immunoprecipitated proteins complexes were analyzed by Western Blot. ChIP experiments and co-immunoprecipitation were repeated at least three times. Primary antibodies are in Suppl. Table 2. Primers for PCR and qRT-PCR analysis of the precipitated chromatin complexes are provided in Suppl. Table 3.

Immunoreactive scores

BCL9 and BCL9-2 scores were calculated for the intensity and percentage of positive epithelial cells as previously described [1; 2]. Staining for nuclear ER and PR were scored according to the Remmele score [3] (IRS 0-12). Membranous Her2 scores were from 0 to 3+. Tissues with scores of 0 and 1 for ER, PR, or Her2 respectively were evaluated as negative, the cases with higher scores as positive, according to the European guidelines for quality assurance in breast cancer screening and diagnosis (4th edition, 2006).

GEO Data analysis

The Gene Expression Omnibus (GEO) GSE6532 dataset [4] with normalized gene expression values of human Affymetrix arrays was retrieved from the NCBI data repository. The dataset was created from early stage patients with ER+ breast cancers and provides information of ER positivity, tamoxifen treatment, and patient's survival. Array data from 263 tamoxifen treated patients were grouped as high or low relative to the median gene expression of BCL9-2 (228065_at).

Statistical methods

Box Plot analyses were performed for the scores of preneoplastic tissue changes and the immunoreactive scores and the significances were calculated using the Mann-Whitney U-test provided by the SPSS package 19. Cumulative hazard for the risk of breast tumors of mice were determined by Kaplan-Meier analysis using SPSS 19. Frequency of mammary tumor development in the mouse models were analyzed by the Chi-Square Test (Suppl. Table 1) and graphs for the dose-response of primary control and BCL9-2 tumor cells were plotted after nonlinear regression analysis using Graph Pad Prism 5. Survival analysis for relapse free patient survival was analyzed by Kaplan-Meier plots and significance was calculated using

the Cox Proportional Hazards Model with the free statistical software R (version 2.14.1). For all other experiments, graphs were calculated as means ± SEM and statistics were evaluated by the Student's t-test using Microsoft Excel. P-values of <0.05 were considered to be statistically significant.

Image acquisition

Immunostains were visualized on a Leica DM5000 B upright microscope with 20x and 40x objectives, colonies from 2D-collagen assays on a Leica DM IRB inverted microscope with a 10x objective and Carmine whole mount stains on a Leica MZ FLIII stereo microscope at 1.25fold magnification (Leica Microsystems). All bright field images were acquired with a DFC290 HD camera with the settings provided by the Leica Application Suite Software LAS Version 3.8. Images of immunofluorescence stains were captured with a DC300 FX camera using Leica Application Suite Software LAS Version 2.8.1 and image overlay processed with the LAS Image Overlay module.

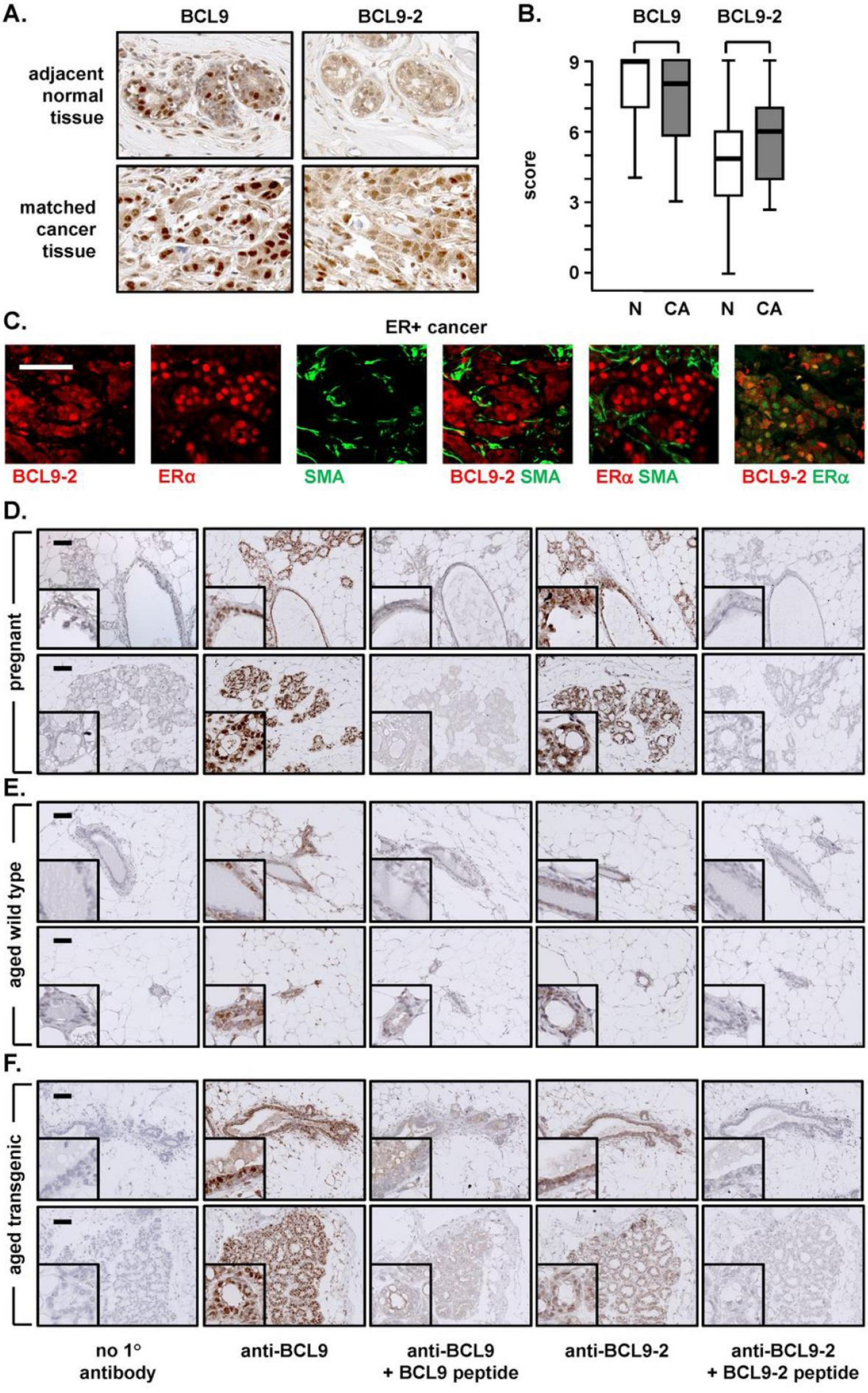
A.
BCL9
BCL9-2
adjacent normal tissue
matched cancer tissue
B.
BCL9
BCL9-2
score
9
6
3
0
N
CA
N
CA
C.
ER+ cancer
BCL9-2
ERα
SMA
BCL9-2 SMA
ERα SMA
BCL9-2 ERα
D.
pregnant
E.
aged wild type
F.
aged transgenic
no 1° antibody
anti-BCL9
anti-BCL9 + BCL9 peptide
anti-BCL9-2
anti-BCL9-2 + BCL9-2 peptide

Supplemental Figure 1: Expression analysis of BCL9 and BCL9-2 in human breast cancers and normal mammary tissues.(A) Example of the immunostains for BCL9 and BCL9-2 on a human breast tissue microarray (BRC481, Pantomics) containing matched specimens of cancer tissues and adjacent normal tissue. **(B)** Box plot analysis for the BCL9 and BCL9-2 immunoreactive scores of the same tissue array from adjacent normal breast tissues (N; n=12) and breast cancer samples (CA; n=24).**(C)** Immunofluorescence stains of BCL9-2, ER and SMA and merged pictures of an additional example for ER+ human breast cancer. **(D-F)** Representative images of immunostains of large ducts (upper panels) and alveoli (lower panels) in the mammary gland from pregnant wild-type C57BL/6 mice (D), 20 month old non-transgenic (E) and age-matched BCL9-2 transgenic females (F). As negative controls immunostains without primary antibody were performed. Tissues were stained with the respective primary BCL9 or BCL9-2 antibodies and competition of the staining was performed with the immunopeptides utilized for antibody production (1 µg peptide/ml antibody solution). Scale bars in the pictures represent 50 µm. Inserts show the staining at higher magnification.

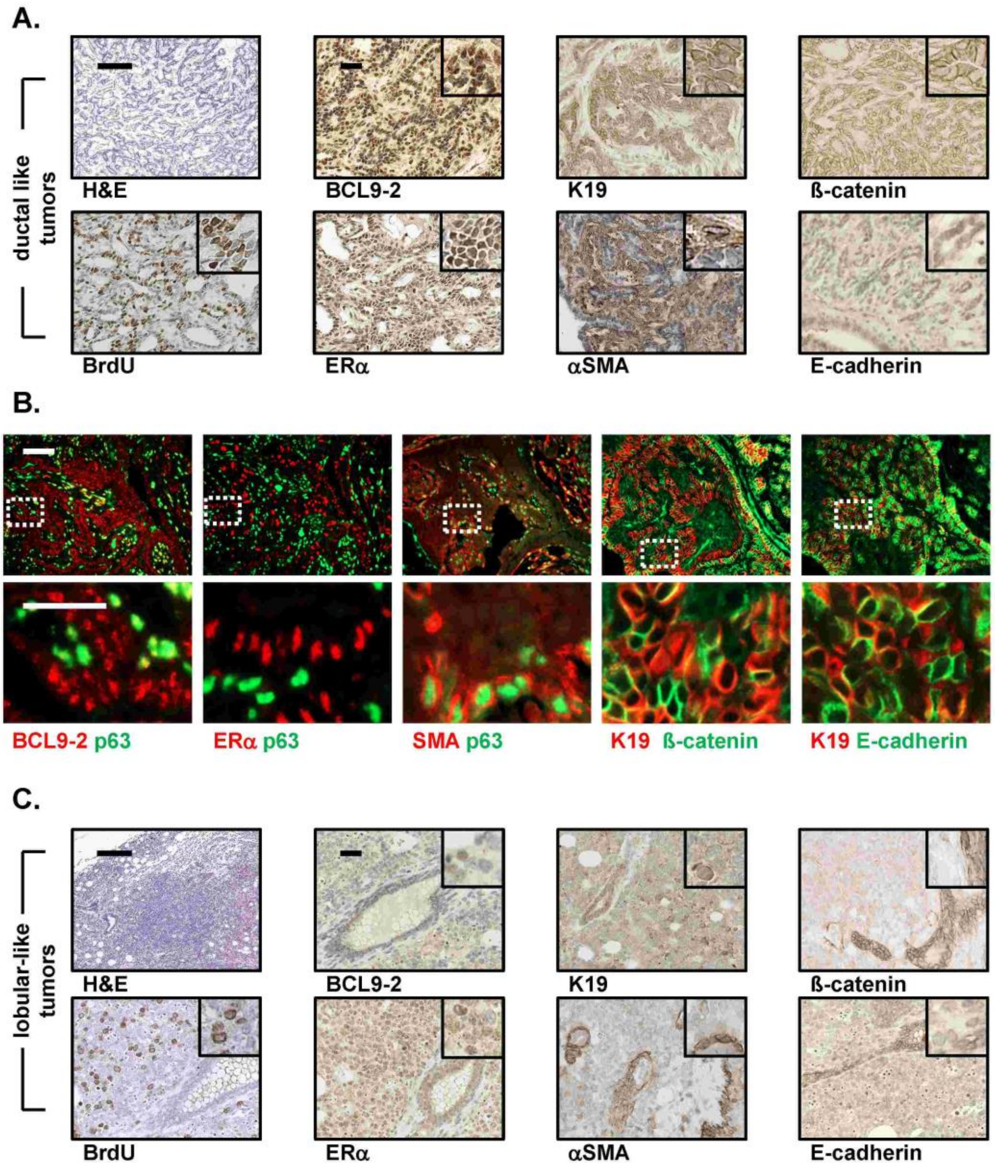

Supplemental Figure 2: Aged BCL9-2 transgenic mice develop ER+ breast cancers.

(A) Additional example for the ductal-like breast tumors from BCL9-2 transgenic female mice. Tissue sections were stained by H&E and with the indicated antibodies for cell specific markers.**(B)** Additional co-immunofluorescence stains for the indicated markers of epithelial and mesenchymal cells in an example of the ductal-like BCL9-2 tumors.**(C)** Example of a lobular-like breast tumor from BCL9-2 transgenic mice. Tissue sections were stained by H&E and with the indicated antibodies.Scale bars in the figures represent 200 µm (H&E) and 50 µm for IHC or IF. Inserts show the staining at higher magnification.

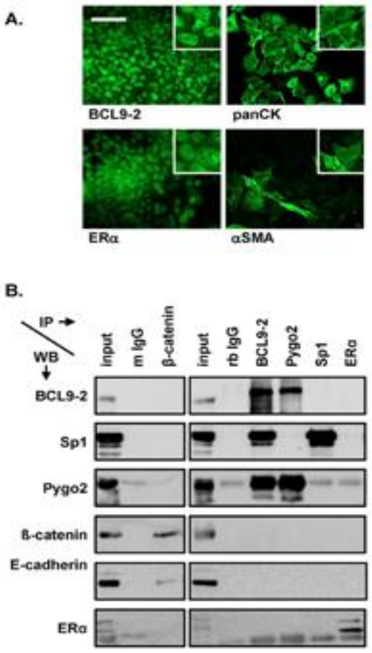

Supplemental Figure 3: Analyses of primary breast tumor cells from BCL9-2 transgenic mice .(A) Additional immunofluorescence stains of primary cells established from mammary tumors of BCL9-2 females. Cells were stained with the indicated antibodies. Scale bar: 50 µm. **(B)** Western Blots (WB) of cell lysates after co-immunoprecipitation (IP) in primary BCL9-2 tumor cells with the indicated antibodies. Mouse and rabbit IgG's were used as negative controls.

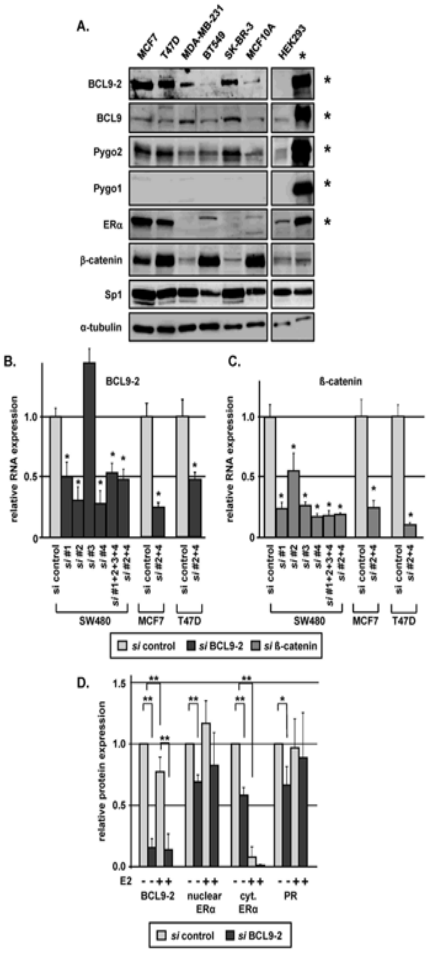

Supplemental Figure 4: Analyses of endogenous BCL9 and Pygo proteins in breast cancer cells and RNA interference against BCL9-2 and ß-catenin. (A) Western Blot analyses for endogenous BCL9/BCL9-2, Pygo1/2, ERα and ß-catenin protein in whole cell lysates obtained from the indicated human breast cancer cell lines. Blots were reprobed with α-tubulin as loading controls. Lysates from wild-type HEK293 cells and transiently transfected for the respective proteins (indicated by an asterisk) were used as negative and positive controls.**(B, C)** RNA interference against BCL9-2 and ß-catenin with On-Target Plus siRNA pools (si 1-4) and the single siRNAs (50 nM) in human cancer cells. Based on the qRT-PCR results, the two most effective siRNAs were further used as pool at a final concentration of 50 nM in all experiments. Graphs show the mean of at least three qRT-PCRs and of their standard error, relative to control siRNA-treated cells. Significant differences are indicated by an asterisk for $P<.05$.**(D)** Densitometry of Western blot analyses from at least

three independent experiments after BCL9-2 knockdown as presented in Figure 6D. The protein levels were normalized to the respective loading controls (Lamin B for nuclear and α-tubulin for cytoplasmatic fractions) and calculated relative to the vehicle treated cells transfected with control siRNAs.

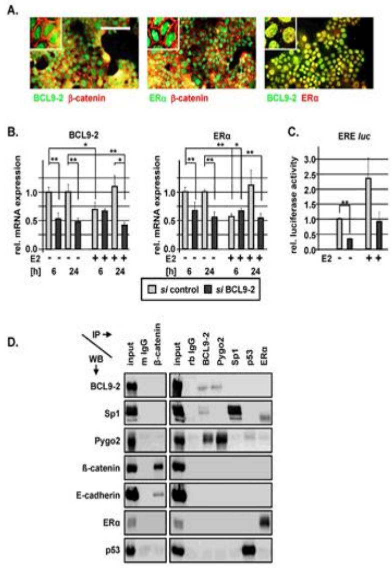

Supplemental Figure 5: BCL9-2 regulates ER expression and interacts with Sp1 in human T47D breast cancer cells. (A) Co-immunofluorescence stains for BCL9-2, ER and ß-catenin in T47D cells. The scale bar represents 50 µm. **(B, C)** The effects of BCL9-2 knockdown on ER expression in T47D breast cancer cells. Cells were pre-treated for 48 hours with the indicated siRNAs followed by hormone starvation overnight. (B) qRT-PCR analysis of the indicated genes and (C) luciferase activity of the ER responsive elements reporter (ERE-luc) in T47D cells after downregulation of BCL9-2 followed by treatment with or without 10 nM estrogen for 6 or 24 hours. * and ** indicates significant differences for *P* <.05 and *P* <.001. Graphs show the mean of at least three independent experiments and of the standard error, relative to control siRNA-treated cells.**(D)** Western Blots (WB) analyses of cell lysates after co-immunoprecipitation (IP) in T47D cells with the indicated antibodies. As control, mouse and rabbit IgG’s were used.

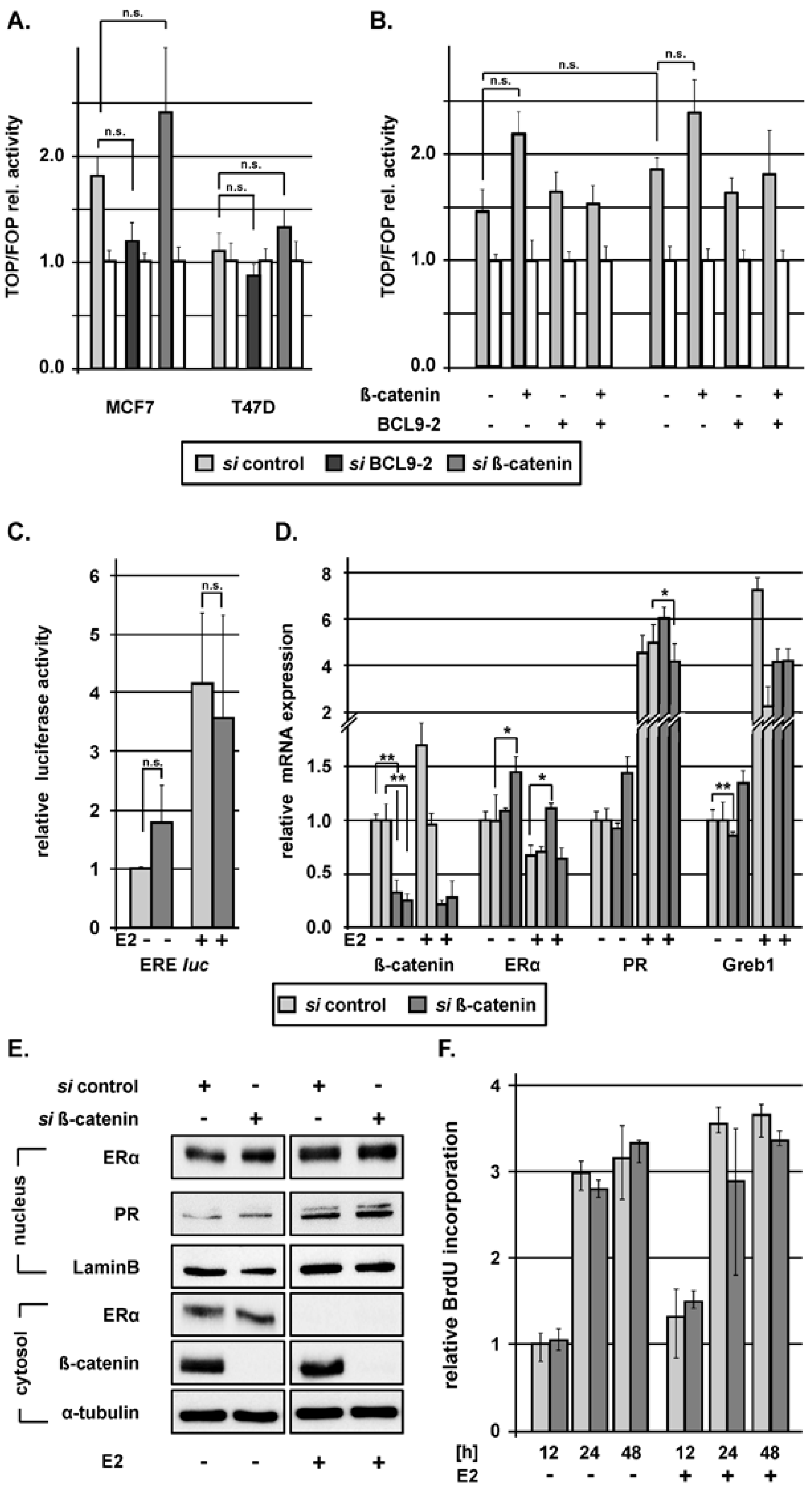

A.
B.
TOP/FOP rel. activity
n.s.
MCF7
T47D
ß-catenin
BCL9-2
si control
si BCL9-2
si ß-catenin
C.
D.
relative luciferase activity
E2
ERE luc
relative mRNA expression
ERα
PR
Greb1
E.
F.
nucleus
cytosol
LaminB
α-tubulin
relative BrdU incorporation
[h]

Supplemental Figure 6: Canonical Wnt/ß-catenin signaling is inactive and β-catenin does not affect ER expression in human ER+ cancer cells. (A, B) Relative luciferase activity of the TOP/FOP Wnt-reporter in MCF7 and T47D cells after downregulation of BCL9-2 and β-catenin (A) and in MCF7 cells after overexpression of BCL9-2 and ß-catenin with and without Wnt 3a stimulation (B). **(C, D)** The effects of β-catenin knockdown on ER expression in MCF7 cells. Cells were pre-treated for 48 hours with the indicated siRNAs, followed by hormone starvation overnight and treatment with 10 nM estrogen (+E2) or vehicle alone (-E2) for additional 6 or 24 hours. Graphs in A-D show the mean of at least three independent experiments and of their standard error, relative to control siRNA-treated cells. Significant differences are indicated with * for $P<.05$ and ** for $P<.01$; n.s. = not significant. Luciferase activity of a reporter containing optimal ER responsive elements (ERE-luc) in MCF7 cells after downregulation of β-catenin followed by treatment with or without estrogen for 6 hours (C). qRT-PCR analysis of the RNA expression of the indicated genes after knockdown of β-catenin followed by treatment with or without estrogen for 6 hours (left bars) and 24 hours (right bars) (D). **(E)** Representative Western Blots of nuclear and cytoplasmatic (cyt.) fractions after β-catenin knockdown and 24 hours of estrogen treatment. Lysates were probed with the indicated antibodies. LaminB1 and alpha-tubulin antibodies were used as loading controls. **(F)** The effects of β-catenin knockdown on proliferation of MCF7 cells as determined by BrdU incorporation assays. Representative time course experiment performed in triplicates and the range for each time point, relative to the indicated control. MCF7 cells were pre-treated with the indicated siRNAs for 48 hours. Cells were further hormone starved overnight, followed by treatment with and without 1 µM E2 for the indicated time points.

Supplemental Table 1:Frequencies of macroscopic mammary tumors of K19-BCL9-2 transgenic females. Development of macroscopic mammary tumors of BCL9-2 animals from different founder lines, virgins and parous animals compared to age matched non-transgenic controls and C57BL/6 controls. Animals were monitored for mammary tumor development until the age of 24 month. Statistic differences for K19-BCL9-2 animals compared to the respective controls were calculated with the one-sided Chi-square test (χ^2) with and without Yates' continuity correction.

**mouse strain**	**animals**	**breast tumors**		χ^2	χ^2 **with Yates' correction**
	n	***n***	***%***	***p***	***p***
non-transgenic controls	***34***	***0***	***0***		
K19-BCL9-2 transgenic (all founder lines)	*109*	***20***	***18***	**0.0035**	**0.0080**
K19-BCL9-2 virgins	*82*	***13***	***16***	**0.0069**	**0.0162**
K19-BCL9-2 parous	*27*	***7***	***26***	**0.0008**	**0.0030**
K19-BCL9-2 line # 1	*18*	***3***	***17***	**0.0071**	**0.0338**
K19-BCL9-2 line # 2	*19*	***4***	***21***	**0.0027**	**0.0125**
K19-BCL9-2 line # 3	*14*	***2***	***14***	**0.0122**	*0.0726*
K19-BCL9-2 line # 4	*25*	***5***	***20***	**0.0032**	**0.0121**
K19-BCL9-2 line # 5	*4*	***1***	***25***	**0.0016**	*0.0962*
K19-BCL9-2 line # 6	*29*	***5***	***17***	**0.0058**	**0.0199**

Supplemental Table 2: Antibodies used in this study for immunohistochemistry (IHC), immunofluorescence (IF), Western Blot (WB), co-immunoprecipitation (IP) and Chromatin-Immunoprecipitation (ChIP).

Antibody	***Host***	***Source***	***Application***
BCL9	*rabbit*	*Brembeck et al., 2011*	*IHC, WB*
BCL9-2	*rabbit*	*A303-152A, Bethyl Lab.*	*IP, ChIP, WB*
BCL9-2	*rabbit*	*Brembeck et al., 2011*	*IHC, IF, WB*
Pygo1	*rabbit*	*Brembeck et al., 2011*	*WB*
Pygo2	*rabbit*	*Brembeck et al., 2011*	*WB, IP, ChIP*
β-catenin	*rabbit*	*Brembeck et al., 2004*	*IHC, IF, WB, IP, ChIP*
β-catenin	*mouse*	*610154, BD Biosciences*	*IF, IP, ChIP*
Flag	*mouse*	*A 8592 M2, Sigma-Aldrich*	*WB*
ERα	*rabbit*	*sc-7207 H-184, Santa Cruz*	*IHC, IF, WB, IP*
ERα	*mouse*	*EI651C0, DCS*	*IF*
panCK	*mouse*	*C2562 C11, Sigma-Aldrich*	*IHC, IF*
E-cadherin	*mouse*	*610182, BD Biosciences*	*IHC, WB*
αSMA	*mouse*	*A2547 1A4, Sigma-Aldrich*	*IHC, IF*
K19	*rabbit*	*ab15463, Abcam*	*IHC*
K19	*rabbit*	*ab76539, Abcam*	*IHC, IF*
P63	*mouse*	*PI006C002, DCS*	*IF*
PR	*rabbit*	*sc-539 C-20, Santa Cruz*	*IHC, WB*
Her2	*rabbit*	*sc-284 C-18, Sigma-Aldrich*	*IHC*
BrdU	*rat*	*ab6326, Abcam*	*IHC*
Cleaved caspase 3	*rabbit*	*9661L, Cell Signaling*	*IHC*
LaminB1	*goat*	*sc-6216 C-20, Santa Cruz*	*WB*
α-tubulin	*mouse*	*T 9026 DM 1A, Sigma-Aldrich*	*WB*
Sp1	*rabbit*	*07-645, Merck Millipore*	*WB, ChIP*
Pol II	*mouse*	*05-623 CTD4H8, Merck Millip.*	*ChIP*
Normal Mouse IgG	*mouse*	*12-371, Merck Millipore*	*ChIP*
p53	*rabbit*	*sc-6243 FL-393, Santa Cruz*	*WB, ChIP, IP*

Supplemental Table 3: Primer sequences used in this study for PCR, quantitative Real-time PCR (qRT-PCR) and cloning. fwd= forward, rev=reverse sequence, h=human, m=mouse, p=promoter.

Primer	*Sequence (5'-3')*	*Application*
transgene fwd	*5'- CAAGGACGACGACGACAAGG*	*PCR*
transgene rev	*5'- CTCTGAATCGAGGGATGGAG*	*PCR*
m+h actin fwd	*5'- GCACAGCTTCTCCTTAATGTCACGC*	*PCR*
m+h actin rev	*5'- TGGCACCACACCTTCTACAATGAGC*	*PCR*
h BCL9-2 fwd	*5'- AATCATGGCAAGACAGGGAATGGCT*	*PCR, qRT-PCR*
h BCL9-2 rev	*5'- TCTTCAGACTTGAGTTGCTAGGCG*	*PCR, qRT-PCR*
h β-catenin fwd	*5'- TTCGAAATCTTGCCCTTTGTCCCG*	*qRT-PCR*
h β-catenin rev	*5'- AATTCGGTTGTGAACATCCCGAGC*	*qRT-PCR*
h ESR1 fwd	*5'- GCATTCTACAGGCCAAATTCA*	*qRT-PCR*
h ESR1 rev	*5'- TCCTTGGCAGATTCCATAGC*	*qRT-PCR*
h PGR fwd	*5'- TCCACCCCGGTCGCTGTAGG*	*qRT-PCR*
h PGR rev	*5'- TAGAGCGGGCGGCTGGAA GT*	*qRT-PCR*
h GREB1 fwd	*5'- GTGGTAGCCGAGTGGACAAT*	*qRT-PCR*
h GREB1 rev	*5'- ATTTGTTTCCAGCCCTCCTT*	*qRT-PCR*
h β-actin fwd	*5'- ATAGCACAGCCTGGATAGCAACGTAC*	*qRT-PCR*
h β-actin rev	*5'- CACCTTCTACAATGAGCTGCGTGTG*	*qRT-PCR*
h pESR1-158+40_fwd	*5'- GGGAAGCTGCTCTTTGGGAT*	*PCR*
h pESR1-158+40_rev	*5'- AAGTGCAGTCCCAGGACGA*	*PCR*
h pESR1-312-130_ fwd	*5'- ACATTAGAGAAAGCCGGCCC*	*PCR*
h pESR1-312-130_rev	*5'- TTGGAGCGATCCCAAAGAGC*	*PCR*
h pESR1-3813-3700 fwd	*5'- CACAGACACGGGGAAGTTGA*	*PCR*

h pESR1-3813-3700 rev	*5'- TTGAATGTGTCTCCAGCCCC*	*PCR*
h pESR1+26+145 fwd	*5'- CTGGGACTGCACTTGCTCC*	*qRT-PCR*
h pESR1+26+145 rev	*5'- GAGGTTAGAGGCGACGCAG*	*qRT-PCR*
h pESR1-268 -195 fwd	*5'- TTAAGCCCAGTCTTCCCTGG*	*qRT-PCR*
h pESR1-268 -195 rev	*5'- AGGCTGAGTTTCACGGCCA*	*qRT-PCR*
h pGAPDH -145+21 fwd	*5'- TACTAGCGGTTTTACGGGCG*	*PCR, qRT-PCR*
h pGAPDH -145+21 rev	*5'- TCGAACAGGAGGAGCAGAGAGCGA*	*PCR, qRT-PCR*
ESR1 WT fwd	*5'- GCGGTACCCCTTTAGCAGATCCTCGTGC*	*cloning*
ESR1 WT rev	*5'- GCTCGAGTGCAGACCGTGTCCCCGCAGG*	*cloning*
ESR1 MT-B fwd	*5'- GCGGTACCCCTTTAGCAGATCCTCGTGCGCCGGAATTCCGGGGCCGTGAAA*	*cloning*
ESR1 MT-A fwd	*5'- CGCAGGCTCCCGCGGAATTCCGCCGGCCAGAGCT*	*cloning*
ESR1 MT-A rev	*5'- AGCTCTGGCCGGCGGAATTCCGCGGGAGCCTGCG*	*cloning*

Supplemental Table 4: Scoring of the preneoplastic changes of the mammary gland.

Score	***Duct dilatation***	***Alveolar differentiation***	***Hyperplastic Alveolar Nodules (size)***	***Score***	***Amount of tissue affected***
0	*none*	*no alveoli*	*None*	***0***	*0%*
1	*moderate*	*moderate*	*< 0,5 mm*	***1***	*< 30%*
2	*strong*	*strong*	*0,5 - 0,75 mm*	***2***	*30% – 60%*
3	*severe*	*severe*	*> 0,75 mm*	***3***	*> 60%*

Supplemental References

1. Brembeck FH, Wiese M, Zatula N, Grigoryan T, Dai Y, Fritzmann J, Birchmeier W. BCL9-2 Promotes Early Stages of Intestinal Tumor Progression. Gastroenterology. 2011; 141: 1359-1370.

2. Sinicrope FA, Ruan SB, Cleary KR, Stephens LC, Lee JJ, Levin B. bcl-2 and p53 oncoprotein expression during colorectal tumorigenesis. Cancer Res. 1995; 55: 237-241.

3. Remmele W and Stegner HE. [Recommendation for uniform definition of an immunoreactive score (IRS) for immunohistochemical estrogen receptor detection (ER-ICA) in breast cancer tissue]. Pathologe. 1987; 8: 138-140.

4. Loi S, Haibe-Kains B, Desmedt C, Lallemand F, Tutt AM, Gillet C, Ellis P, Harris A, Bergh J, Foekens JA, Klijn JG, Larsimont D, Buyse M et al. Definition of clinically distinct molecular subtypes in estrogen receptor-positive breast carcinomas through genomic grade. J. Clin. Oncol. 2007; 25: 1239-1246.
